# Lung cancer cell-intrinsic IL-15 promotes cell migration and sensitizes murine lung tumors to anti-PD-L1 therapy

**DOI:** 10.1186/s40364-024-00586-w

**Published:** 2024-04-19

**Authors:** Shaojie Hu, Kelin Meng, Tianlai Wang, Rirong Qu, Boyu Wang, Yu Xi, Taiyan Yu, Zhiwei Yuan, Zihao Cai, Yitao Tian, Chenxi Zeng, Xue Wang, Wenbin Zou, Xiangning Fu, Lequn Li

**Affiliations:** Thoracic Surgery Laboratory, Department of Thoracic Surgery, Tongji Hospital, Tongji Medical College, Huazhong University of Science and Technology, 1095 Jie Fang Avenue, 430030 Wuhan, Hubei China

**Keywords:** Lung adenocarcinoma, IL-15, Metastasis, Immunotherapy

## Abstract

**Background:**

IL-15 plays a vital role in enhancing NK cell- and T-cell-mediated antitumor immune responses; however, the direct effect of IL-15 on tumor cells has not been fully elucidated. Herein, we investigated the effect of IL-15 on lung adenocarcinoma cells.

**Methods:**

Silencing and overexpression techniques were used to modify endogenous IL-15 expression in tumor cells. Transwell assays were used to assess tumor cell migration and invasion; a live-cell analysis system was used to evaluate cell motility; cellular morphological changes were quantified by confocal fluorescence microscopy; the molecular mechanisms underlying the effect of IL-15 on tumor cells were analyzed by western blotting; and RhoA and Cdc42 activities were evaluated by a pulldown assay. NCG and C57BL/6 mouse models were used to evaluate the functions of IL-15 in vivo.

**Results:**

Cancer cell-intrinsic IL-15 promoted cell motility and migration in vitro and metastasis in vivo via activation of the AKT-mTORC1 pathway; however, exogenous IL-15 inhibited cell motility and migration via suppression of the RhoA-MLC2 axis. Mechanistic analysis revealed that both the intracellular and extracellular IL-15-mediated effects required the expression of IL-15Rα by tumor cells. Detailed analyses revealed that the IL-2/IL-15Rβ and IL-2Rγ chains were undetected in the complex formed by intracellular IL-15 and IL-15Rα. However, when exogenous IL-15 engaged tumor cells, a complex containing the IL-15Rα, IL-2/IL-15Rβ, and IL-2Rγ chains was formed, indicating that the differential actions of intracellular and extracellular IL-15 on tumor cells might be caused by their distinctive modes of IL-15 receptor engagement. Using a Lewis lung carcinoma (LLC) metastasis model, we showed that although IL-15 overexpression facilitated the lung metastasis of LLC cells, IL-15-overexpressing LLC tumors were more sensitive to anti-PD-L1 therapy than were IL-15-wild-type LLC tumors via an enhanced antitumor immune response, as evidenced by their increased CD8^+^ T-cell infiltration compared to that of their counterparts.

**Conclusions:**

Cancer cell-intrinsic IL-15 and exogenous IL-15 differentially regulate cell motility and migration. Thus, cancer cell-intrinsic IL-15 acts as a double-edged sword in tumor progression. Additionally, high levels of IL-15 expressed by tumor cells might improve the responsiveness of tumors to immunotherapies.

**Supplementary Information:**

The online version contains supplementary material available at 10.1186/s40364-024-00586-w.

## Introduction

Immune checkpoint blockade (ICB) therapies improve the antitumor immune response against cancer cells and have become standard treatments for non-small cell lung cancer (NSCLC). However, most patients with NSCLC do not benefit from ICB therapy [1, 2]. Currently, multiple therapeutic approaches have been developed to remodel the immunosuppressive microenvironment, aiming to more effectively reinvigorate TILs to improve antitumor immunotherapy outcomes. Among these new approaches is treatment with agonists of cytokines, which are potent immunomodulatory factors that can effectively enhance the effector functions of TILs, including their cytolytic activity and proliferative capability [3, 4]. Interleukin (IL)-15, a cytokine that belongs to a family of interleukins that use the common cytokine receptor γ-chain, is essential for the proliferation and effector functions of NK and CD8^+^ T cells [5–7]. Due to their robust effect on the activation of immune cells, IL-15 and IL-15 agonists are considered promising antitumor agents [6, 8–10].

IL-15 is produced mainly by macrophages, monocytes, and dendritic cells and is expressed in certain cells, including epithelial cells, fibroblasts, keratinocytes, and nerve cells [11–13]. IL-15 utilizes three distinct receptor chains bind to target cells. The IL-15 receptor complex consists of an IL-15 receptor alpha subunit (IL-15Rα), an IL-2 receptor beta subunit (IL-2Rβ), and a gamma subunit (IL-2Rγ). IL-15Rα is by itself a specific receptor with a high affinity for IL-15 [14]. In lymphocytes, the binding of IL-15 to its receptor complex enables the activation of the JAK/STAT, PI3K-AKT, and MAPK pathways [15]. The antitumor effect of IL-15 on the immune system is well documented [16]. We previously showed that CD8^+^CD57^+^ T cells in tumors exhibit an inferior response to PD-1 blockade compared to that of their CD8^+^CD57^−^ counterparts, and interestingly, IL-15 preferentially restored the effector function of these cells [17].

Although the pivotal role of IL-15 in enhancing NK cell- and T-cell-mediated antitumor immune responses is well recognized, few studies have investigated the direct effect of IL-15 on tumor cells [18, 19]. Khawam et al. reported that human renal cancer cells express membrane-bound IL-15 and respond to the soluble IL-15 receptor α chain, leading to epithelial-to-mesenchymal transition (ΕΜΤ) [20]. ΕΜΤ has been suggested to play a crucial role in metastasis [21]. Recently, the existence of an intermediate state of EMT in which cancer cells incompletely lose epithelial markers and incompletely gain mesenchymal marker expression has been recognized, and more importantly, cancer cells in this partial EMT state have more aggressive phenotypic characteristics than those that have completed EMT [22–25].

In this study, we found that IL-15 was expressed by lung cancer cells. We investigated the biological function of cancer cell-intrinsic IL-15 and its underlying mechanism. Intracellular IL-15 in cancer cells was involved in regulating cell morphology and promoting cell motility and migration in vitro by activating ATK-mTORC1 signaling. However, exogenous IL-15 did not activate ATK-mTORC1 signaling but rather downregulated RhoA activity and led to the inhibition of cell migration and invasion. Despite promoting cell migration via intracellular IL-15, a high level of IL-15 expression in cancer cells enhances the effector function of T cells and sensitizes tumors to anti-PD-L1 therapy. Thus, our studies imply that endogenous IL-15 in cancer cells plays a pivotal role in the progression of cancer and is an indicator of the responsiveness of tumors to ICB therapy.

## Materials and methods

### Cell lines and cell culture

The human lung adenocarcinoma A549 (ATCC Cat# CRL-7900), HCC827 (KCLB Cat# 70,827), H1975 (ATCC Cat# CRL-5908), H2030 (ATCC Cat# CRL-5914), H23 (ATCC Cat #CRL-5800), and PC9 (BCRJ Cat #0331) cell lines were obtained from Cobioer Biosciences (Nanjing, China). Short tandem repeat (STR) analyses of the A549, HCC827, and H1975 cell lines were performed in 2017, and STR analyses of the H2030 and PC9 cell lines were performed in 2019. A549 cells were maintained in F12K medium (Boster, CA, USA) supplemented with 10% fetal bovine serum (FBS) (Gibco, Grand Island, NY, USA), 100 U/mL penicillin, and 0.1 mg/mL streptomycin (HyClone, Logan, UT, USA). The remaining cell lines were cultured in RPMI 1640 medium (HyClone, Omaha, NE, USA). The LLC cell line (ATCC Cat #CRL-1642) was maintained in DMEM (HyClone) supplemented with 10% FBS. All cell lines were incubated at 37 °C in a humidified incubator with 5% CO_2_. All cell lines were confirmed to be *Mycoplasma*-negative (Biothrive Sci. & Tech. Ltd., Shanghai, China), and the cell lines were used within 3 months after resuscitation.

### Antibodies and reagents

All antibodies and reagents are listed in Table S1.

### Patients and tissue samples

Lung adenocarcinoma specimens and normal lung tissue specimens were obtained from patients who underwent pulmonary lobectomy resection prior to radiation or chemotherapy between 2019 and 2022 in the Department of Thoracic Surgery, Tongji Hospital. Pathology was used to diagnose the tumors based on the criteria of the World Health Organization (WHO). The tumor-node-metastasis (TNM) stage was determined according to the guidelines of the American Joint Committee on Cancer Staging Manual, 8th edition. The use of human tissue specimens was approved by the Institutional Ethics Committee of the Huazhong University of Science and Technology.

### Small interfering RNA (siRNA) transfection

siRNA sequences targeting IL-15, IL-15Rα, and vimentin were synthesized by RiboBio (Guangzhou, China). The targeted sequences are shown in Table S2. siRNAs (50 nmol/L) and Lipofectamine 3000 (Invitrogen, Carlsbad, CA, USA) were gently mixed in serum-free medium according to the manufacturer’s instructions. The transfection mixture was added to the culture plate, and subsequently, cell suspensions were seeded and cultured for 48 h. The knockdown efficiency of the siRNAs was evaluated via immunoblot analysis.

### Plasmid construction

The pCDH-EF1-copGFP plasmid and pHAGE-puro plasmid were purchased from GenePharma (Shanghai, China). The human IL-15 overexpression plasmid was purchased from Genomeditech (Shanghai, China). For construction of the mouse IL-15 overexpression vectors, targeted primers were designed to amplify the open reading frame (ORF) of mouse IL-15 using 2 × Phanta Max Master Mix (Cat: P515-01; Vazyme, Nanjing, China) (Table S2). Mlu-I and Bmt-I (Thermo Fisher Scientific, USA) were used to digest the pHAGE-puro plasmid. The mouse IL-15-Flag (ORF) sequence was ligated into the resultant plasmid. Primers targeting human IL-15 were designed to construct the short hairpin RNA (shRNA)-IL-15 plasmid (Table S2). The pCDH-EF1-copGFP plasmid was digested with Age I and EcoR I (Thermo Fisher Scientific, USA). The constructed plasmid was subsequently transformed into DH5α cells (Tsingke, Wuhan, China), after which the resulting constructs were sequenced.

### Transfection and retroviral infection

Polyplus transfection reagent (Cat:101,000,046, jetPRIME, France) was used to transfect the constructed plasmids, along with the packaging plasmid, psPAX2, the envelope plasmid, and pMD2.G. The packaging cell line (293T cells) was cultured for 48 h. The lentivirus-containing culture supernatants were used to infect the target cells for up to 6 h. The transduced cells were selected by incubation with puromycin-containing culture medium for up to 2 weeks.

### Western blot analysis

Cells were lysed using RIPA lysis buffer (50 mM Tris (pH 7.4), 150 mM NaCl, 1% Triton X-100, 1% sodium deoxycholate, and 0.1% SDS. Proteins were separated using sodium dodecyl sulfate-polyacrylamide gel electrophoresis (SDS-PAGE) and transferred onto Immobilon®-P membranes (Merck Millipore, Darmstadt, Germany). An enhanced chemiluminescence (ECL) detection system (Tanon 5200, Shanghai, China) was used to visualize the protein bands.

### RNA isolation and reverse transcription-quantitative PCR (RT‒qPCR) analysis

TRIzol (Takara Bio Inc, Shiga, Japan) was used to extract total RNA from the cells. cDNA was reverse transcribed from mRNA using an RT Reagent Kit (Vazyme). The primers used were purchased from Tsingke Biotechnology and are listed in Table S2. Target mRNA expression was assessed by quantitative PCR with a SYBR Green Master Mix Kit (Vazyme) and an Applied Biosystems thermal cycler (Vilnius, Lithuania). In addition, negative control reactions without template DNA were included for every experiment. All the reactions were performed in triplicate. The relative expression of target genes was determined by the 2^−ΔΔCt^ method.

### Cell viability assay

Cell viability was assessed by using a CCK-8 kit (Abbkine, Wuhan, China). Cells were seeded in 96-well plates (3,000 cells/well). The absorbance was measured at 450 nm using a microplate reader (Tecan, Baldwin Park, CA, USA).

### Transwell migration and invasion assays

For analysis of cell migration, a Transwell chamber with an 8 μm pore size was used. The cells were resuspended in serum-free culture medium and seeded into the upper compartment (50,000 cells/100 µL/well). Medium supplemented with 10% FBS was added to the lower compartment as a chemoattractant. During the 24 h incubation period, the cells migrated through the pores of the membrane and reached the bottom surface of the chamber. The migrated cells were fixed with 4% formaldehyde, stained with 0.1% crystal violet and then observed under a microscope at a magnification of 200×. Five random fields of view were selected. The number of migrated cells was determined and averaged to measure cell migration.

For analysis of the invasive capability of the cells, a layer of Matrigel (Corning, Bedford, MA, USA) was applied to precoat the upper surface of the membrane in the upper chamber, and an invasion assay was performed following a procedure similar to that described above for the cell migration assay.

### Cdc42 activation assay

A Cdc42 Activation Assay Kit (New East Biosciences, PA, USA) was used to assess the activation of Cdc42 proteins. IL-15 overexpressing cells were treated with LY294002 (MedChemExpress, NJ, USA) or rapamycin (MedChemExpress, NJ, USA) for 24 h and were subsequently harvested and lysed. The cell lysates were incubated with a conformation-specific anti-Cdc42-GTP antibody. Then, GTP-bound Cdc42 was pulled down by protein A/G agarose and analyzed by immunoblotting with an anti-Cdc42 antibody.

### RhoA activation assay

The cells were starved for 6 h and then treated with IL-15. After 24 h, the cells were harvested and lysed. A GST fusion protein expressing the Rho-binding domain of human Rhotekin (Cytoskeleton, Denver, CO, USA) was used to specifically bind GTP-bound RhoA. The pulldown assay was performed according to the manufacturer’s protocol.

### Cell motility assay

Cells were seeded in a 96-well plate and subjected to the indicated treatment. Images of the cells were acquired in a live-cell analysis system (Sartorius, Göttingen, Germany) at 15-min intervals over a total imaging duration of 24 h. The time-lapse images were then compiled into a video, and cell motility was analyzed using the Manual Track plugin of ImageJ. The analyzed data were imported into the Chemotaxis and Migration Tool ImageJ plugin (ibidi), and the distance and speed were calculated.

### Isolation of peripheral blood mononuclear cells (PBMCs)

Peripheral blood was collected and stored in collection tubes containing K2 EDTA as an anticoagulant. Human lymphocyte separation medium (Dakewe, Shenzhen, China) was used to isolate PBMCs via density gradient centrifugation.

### Culture of patient-derived lung cancer explants

Tumor tissues were obtained from patients who underwent pulmonary lobectomy in the Thoracic Surgery Department of Tongji Hospital. The lung cancer patients did not undergo radiotherapy or chemotherapy before surgery. After fresh tumor tissues were obtained, a portion of the tumor tissue was fixed and sliced, while the remaining portion was carefully cut into small pieces with a volume of 8 mm^3^ and cultured in medium containing 10% FBS. An anti-PD-1 mAb (10 µg/mL) (Biolegend, San Diego, CA, USA) was added to the cultures, which were incubated for 24 h. Subsequently, single-cell suspensions were prepared and then filtered through a mesh with a diameter of 70 μm. The single-cell suspensions were centrifuged through 40% and 70% Percoll (GE Healthcare, Uppsala, Sweden) density gradients. Lymphocytes were collected and stained for the indicated cell surface and intracellular markers. The use of human tumor tissue was approved by the Ethics Committee of Huazhong University of Science and Technology.

### Immunoprecipitation (IP)

The cells were lysed using GST lysis buffer (10% glycerol, 1% NP40, 50 mM Tris-HCl (pH 7.8), 150 mM NaCl, and 2 mM MgCl_2_) supplemented with a complete protease and phosphatase inhibitor cocktail. The samples were incubated with 5 µg of a primary antibody overnight at 4 °C before adding Pierce ^TM^ protein A/G magnetic beads (MedChemExpress, NJ, USA) for an additional 1 h of incubation. A magnetic stand was used to collect the magnetic beads, and the bound proteins were eluted and subsequently analyzed by immunoblotting.

### Phalloidin and vinculin staining and imaging by confocal microscopy

Cells were seeded onto coverslips and treated with the indicated reagents. After treatment, the cells were fixed with 4% paraformaldehyde in phosphate-buffered saline (PBS) for 15 min. Subsequently, the cells were permeabilized with 0.3% Triton X-100 (Standard Reagent, Hyderabad, India) in PBS for 10 min. The cells on the cover slips were incubated with iFluor™ 647-labeled phalloidin (Yeasen, Shanghai, China) for 90 min and DAPI (Servicebio, Wuhan, China) for 20 min. The stained cells were visualized and imaged by a confocal microscopy system (Olympus FV1000, Tokyo, Japan). The filopodia are slender, elongated protrusions ranging in length from 1 to 10 μm. They are characterized by their thin and straight structure and lack of a visible head or bulbous enlargement [26]. In each experiment, ten random images were selected from each group, and the filopodia in each image were counted to calculate the average number. For quantification of stress fiber formation, the boundary of the cells was determined by using differential interference contrast images, after which the total intensity of the phalloidin fluorescence was calculated by ImageJ software and used as a measure of stress fiber formation [27]. After permeabilization, the sections were stained with vinculin polyclonal antibody (Proteintech, Wuhan, China) and Alexa488-conjugated or Cy5-conjugated anti-rabbit IgG antibody (Servicebio, Wuhan, China). The number of focal adhesions in the cells was counted by using ImageJ software.

### Immunohistochemical staining and quantification

Normal lung tissues and tumors were fixed with 4% paraformaldehyde for 48 h. Following fixation, the samples were dehydrated, embedded in paraffin, and sliced into 5-µm-thick sections. Deparaffinization and rehydration of the sections were performed. Antigen retrieval was carried out using an Antigen Retriever system (Prestige Medical, England) by incubating the sections with EDTA buffer (pH = 9) for 15 min followed by cooling at room temperature for 30 min. To block endogenous peroxidase activity, the sections were treated with 3% hydrogen peroxide for 10 min. After washing with PBS, the sections were incubated with primary antibodies against IL-15 or CD8 for 12 h at 4 °C (Table S1). The sections were then incubated with horseradish peroxidase (HRP)-conjugated secondary antibodies. Immunoreactivity was visualized using diaminobenzidine (DAB) staining (GK600705; GeneTech, Shanghai, China). Slides not incubated with primary antibodies served as negative controls. The average integrated optical density (average IOD) of IL-15 was measured via IHC staining to assess the immunostaining intensity of IL-15 in lung cancer tissue and normal lung tissue. Three tumor cell regions were measured (400× magnification, Zeiss microscope), and the average IOD of IL-15 in each patient was calculated as the mean value of three images. Image-Pro Plus software was used to analyze the images. In brief, when images were opened with Image-Pro Plus software, the average IOD of IL-15 was calculated automatically using the HSI threshold (25, 255, 255). The density of CD8^+^ T cells was determined by selecting four independent areas at low magnification. Subsequently, three random microscopic fields were selected at a higher magnification from each area. The number of CD8^+^ T cells in the tumor was determined by counting in 12 high-power microscopic fields, and the density of CD8^+^ T cells was calculated as the average count across these 12 high-power fields.

### Animal models

NCG mice and C57BL/6 mice (male, 6 to 8 weeks old) were obtained from GemPharmatech Co. Ltd. (Nanjing, China). The mice were housed in pathogen-free facilities. All animal experiments were conducted in accordance with the Guidelines for the Care and Use of Laboratory Animals of Tongji Hospital (Wuhan, China).

#### Tumor growth model

C57BL/6 mice were inoculated subcutaneously with 1 × 10^6^ LLC cells transduced with Lv-mCtrl (*n* = 7) or Lv-mIL-15 (*n* = 7) in the right flank. Seven days after the subcutaneous injection of LLC cells into the mice, a local nodule had formed. The tumor volume was measured with calipers every two days. The tumor volume was calculated with the following equation: tumor volume (mm^3^) = length × width^2^ × 0.5. Mice were euthanized when the tumor volume reached ≈ 2000 mm^3^.

#### Tumor metastasis model

A total of 1 × 10^6^ A549-shRNA-control cells (*n* = 3), A549-shRNA-IL15 cells (*n* = 3), A549-Lv-hCtrl cells (*n* = 3), and A549-Lv-hIL-15 cells (*n* = 3) were resuspended separately in 100 µL of PBS and intravenously injected into NCG mice via the tail vein. Each group contained 3 mice. After 7 days, the NCG mice were euthanized, and their lungs were harvested. The lung tissue was fixed with 4% paraformaldehyde, embedded in paraffin, and sliced into 5-µm-thick sections. Hematoxylin and eosin (HE) staining was performed on the sections, and the metastatic foci were observed under a microscope (Zeiss LSM 780, Germany). The metastatic areas were analyzed in randomly acquired images and quantified using ImageJ. Similarly, 1 × 10^6^ Lv-mCtrl-LLC cells or Lv-mIL-15-LLC cells were resuspended separately in 100 µL of PBS and injected into C57BL/6 mice via the tail vein. After 3, 5, and 7 days, the metastatic areas were analyzed.

#### Immunotherapy model

Lv-mCtrl-LLC cells (1 × 10^6^ cells/mouse, *n* = 3) and Lv-mIL-15-LLC cells (1 × 10^6^ cells/mouse, *n* = 5) were injected into C57BL/6 mice via the tail vein. Subsequently, the mice were divided into four groups: the Lv-Ctrl group treated with isotype control (i.p., 10 mg/kg), the Lv-Ctrl group treated with an anti-PD-L1 antibody (i.p., 10 mg/kg), the Lv-mIL-15 group treated with isotype control, and the Lv-mIL-15 group treated with an anti-PD-L1 antibody. Treatment with isotype control or the anti-PD-L1 antibody was initiated on day 5 after injection of tumor cells. The isotype control and anti-PD-L1 antibodies were administered on day 0, day 3, and day 6. On day 9 after antibody treatment, the mice were euthanized, and their lungs were harvested, fixed, sliced, and stained with HE. The metastatic area was analyzed using ImageJ.

C57BL/6 mice were inoculated subcutaneously with 1 × 10^6^ LLC cells transduced with Lv-mCtrl (*n* = 7) or Lv-mIL-15 (*n* = 7) in the right flank. Seven days after the subcutaneous injection of LLC cells into the mice, a local nodule had formed. The Lv-Ctrl group and Lv-mIL-15 group treated with an anti-PD-L1 antibody (i.p., 10 mg/kg). Treatment with the anti-PD-L1 antibody was initiated on day 7 after injection of tumor cells. The anti-PD-L1 antibodies were administered on day 0, day 3, and day 6. On day 12 after antibody treatment, the mice were euthanized. The tumor volume was measured with calipers every two days. The tumor volume was calculated with the following equation: tumor volume (mm^3^) = length × width^2^ × 0.5. Mice were euthanized when the tumor volume reached ≈ 2000 mm^3^.

### Flow cytometry

Tumor tissues were cut into small pieces, minced, suspended in PBS, and filtered through 100 μm nylon cell strainers (Biosharp, Anhui, China). Lymphocytes were isolated and enriched using mouse lymphocyte separation medium (Dakewe, Shenzhen, China) following centrifugation (800 × *g*, 10 min). Cell surface staining was performed using antibodies against CD8, CD3, and CD45 (Table S1). Dead cells were excluded by positive labeling with Fixable Viability Dye eFluor™ 780 (eBioscience, Waltham, Massachusetts, USA). For intracellular staining, after surface staining, lymphocytes were fixed using Foxp3/Transcription Factor Fixation/Permeabilization Buffer (eBioscience) and permeabilized using permeabilization buffer (eBioscience) before intracellular staining with antibodies against Ki67 and perforin (Table S1). To determine the effect of the IL15/IL15Rα complex (MedChemExpress) on CD3^+^ T cells, human peripheral blood mononuclear cells (PMBCs) were used. PBMCs were stained with antibodies against CD3, CD4, GZMB, Ki67, and perforin after 3 days of stimulation with the IL15/IL15Rα complex. Flow cytometric analysis was conducted using an Attune NxT acoustic flow cytometer (Thermo Fisher Scientific, Waltham, Massachusetts, USA). The data were analyzed using FlowJo v10 software (BD Biosciences).

### Analysis of IL-15 with ELISA

Tumors were freshly harvested and immediately centrifuged at 4 °C for 10 min at 106× *g* to obtain tumor interstitial fluid [28]. The concentration of IL-15 in the tumor interstitial fluid was assessed by the enzyme-linked immunosorbent assay (ELISA) according to the manufacturer’s guidelines (Thermo Fisher Scientific, Cranbury, NJ, USA). To determine the amount of IL-15 in the cell culture supernatants and cell lysates, 1 × 10^6^ IL-15-overexpressing A549 cells and an equal number of control A549 cells were cultured for 24 h. The supernatants were collected, and the cells were lysed with 500 µL of cell lysis buffer (0.5% NP-40 in PBS). IL-15 was detected by ELISA according to the manufacturer’s instructions (BioLegend). Similar methods were used to detect IL-15 in the cell lysates and culture supernatants from IL-15-overexpressing LLC cells and control LLC cells.

### Kaplan–Meier plotter analysis

Kaplan–Meier Plotter (KM plotter, https://kmplot.com/analysis/) was used to analyze the prognostic significance of IL-15 mRNA expression. To analyze the prognostic value of IL-15 in patients with lung adenocarcinoma, the patients were divided into two cohorts based on the mRNA level of IL-15. Survival was compared between the two patient cohorts through the construction of Kaplan-Meier survival curves. The construction of the Kaplan-Meier survival curves included determining hazard ratios along with the corresponding 95% confidence intervals, as well as the log-rank *p* values.

### Statistical analysis

Statistical analysis and graphing were performed using GraphPad Prism software v8.0 (GraphPad Prism, RRID: SCR_002798). The data in the bar graphs are presented as the means ± standard deviations (SDs). An unpaired two-tailed Student’s *t* test was used for comparisons between two groups. One-way analysis of variance (ANOVA) was used for comparisons among more than two groups. To assess differences in tumor volume between the treatment groups, two-way ANOVA followed by Sidak’s multiple comparison test was performed. Statistical significance is indicated as follows: * *p* < 0.05, ** *p* < 0.01, *** *p* < 0.001, and **** *p* < 0.0001.

## Results

### Cancer cell-intrinsic IL-15 promotes cell motility, migration, and invasion

**Fig. 1:**
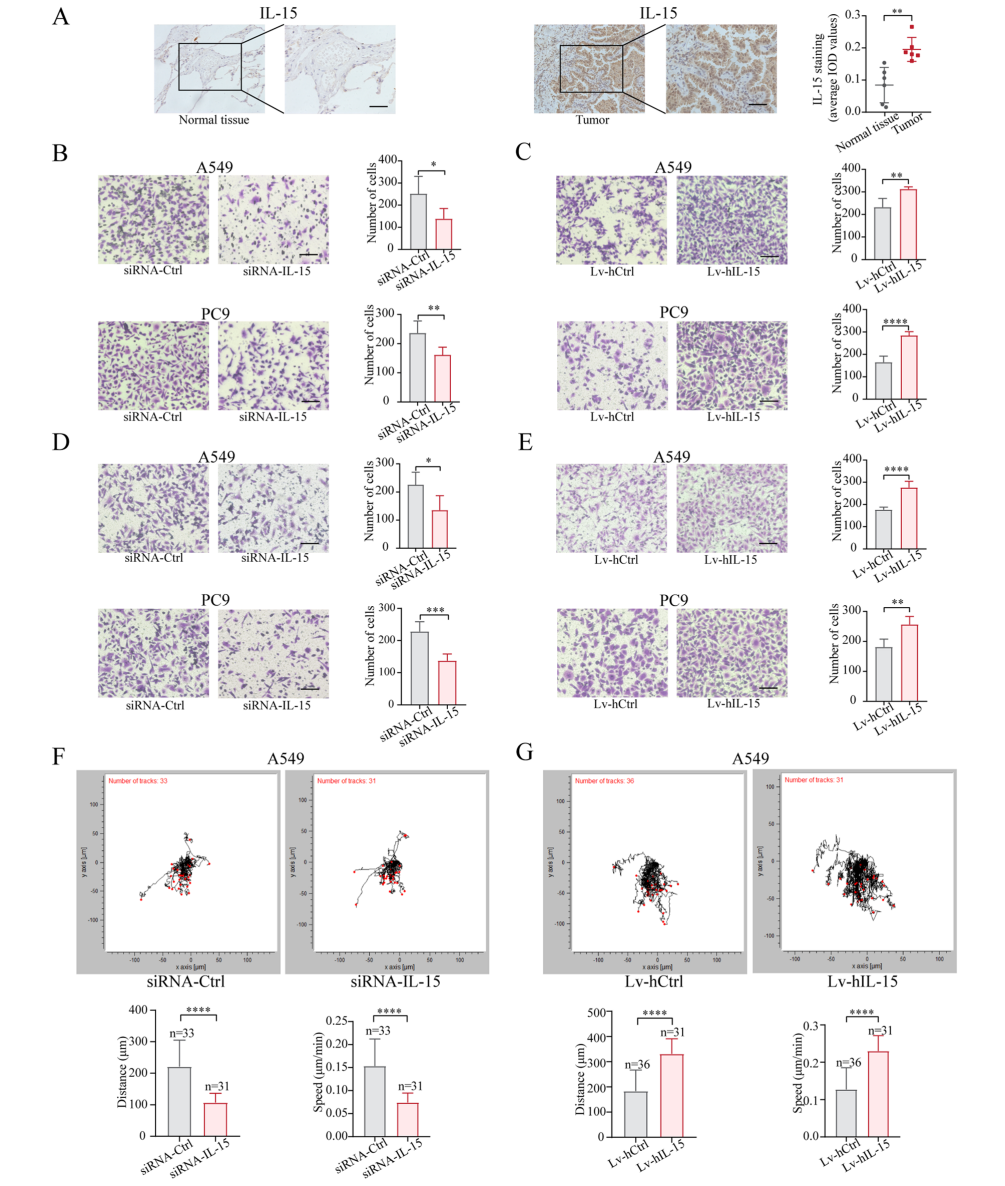
Cancer cell-intrinsic IL-15 promotes cell migration, invasion, and motility. (**A**), IHC staining of IL15 in lung adenocarcinoma tissue and corresponding normal lung tissue (*n* = 6). Scale bar, 50 μm. **, *p* < 0.01. (**B-E**), Transwell migration (B and C) and invasion (D and E) assays were performed on A549 cells and PC9 cells transfected with siRNA-IL15 and siRNA-Ctrl (B, D) or with Lv-hIL-15 and Lv-hCtrl (C, E). Scale bar, 100 μm. *, *p* < 0.05; **, *p* < 0.01; ***, *p* < 0.001; ****, *p* < 0.0001. (**F and G**), A549 cells were transfected with siRNA-IL15 or siRNA-Ctrl for 48 h, and subsequently, the motion trajectory of the cells was recorded using a live-cell analysis system for 24 h (F). Alternatively, the motion trajectories of A549 cells transduced with Lv-hIL-15 or Lv-hCtrl were recorded for 24 h (G). The motion images were analyzed using manual tracking in ImageJ with the Chemotaxis and Migration Tool plugin. The data in the bar graphs are presented as the means ± SDs and were analyzed with two-tailed Student’s *t* test. ****, *p* < 0.0001

IL-15 is expressed predominantly by myeloid cells [29, 30]. However, few studies have shown that IL-15 can be expressed by tumor cells, except for human renal cancer cells [20, 31, 32]. As shown in Fig. 1A, IL-15 was expressed by lung adenocarcinoma cells as determined by immunohistochemistry, and surprisingly, the expression levels of IL-15 in tumors were significantly greater than those in normal lung tissue. Moreover, IL-15 was also expressed by lung adenocarcinoma cell lines (Figure S1A).

Next, we used loss- and gain-of-function approaches to assess the biological function of endogenous IL-15 in cancer cells. As IL-15 promotes the proliferation of NK cells and T cells [33], we determined whether cancer cell-intrinsic IL-15 increases the proliferation of tumor cells. To this end, A549 and PC9 cells were transfected with a siRNA specific for IL-15 (siIL-15) or transduced with the pGMLV-PA6-IL-15 vector (Lv-hIL-15) or corresponding control vector (Lv-hCtrl). The expression level of IL-15 in tumor cells did not affect their proliferation (Figure S1B). Metastasis is a hallmark of cancer, and the migration and invasion abilities of tumor cells are integral components of metastasis. We therefore examined whether cancer cell-intrinsic IL-15 affects cell migration and invasion in vitro. The migratory capabilities of A549 and PC9 cells were significantly decreased after transfection with siRNA-IL15 compared to those after transfection with siRNA-Ctrl (Fig. 1B). Conversely, the overexpression of IL-15 in A549 and PC9 cells led to an increase in cell migration compared to that in cells transduced with Lv-hCtrl (Fig. 1C). Similar observations were obtained in the H1975 and H2030 cell lines (Figure S1C and D). Moreover, knockdown of IL-15 significantly suppressed cell invasion (Fig. 1D), whereas overexpression of IL-15 enhanced cellular invasion (Fig. 1E). Additionally, compared with control treatment, knockdown of IL-15 reduced but overexpression of IL-15 increased the motility of tumor cells (Fig. 1F and G). Our data showed that cancer cell-intrinsic IL-15 promotes cell motility, migration, and invasion in vitro but does not alter cell proliferation.

### Overexpression of IL-15 in tumor cells facilitates the formation of metastases

**Fig. 2:**
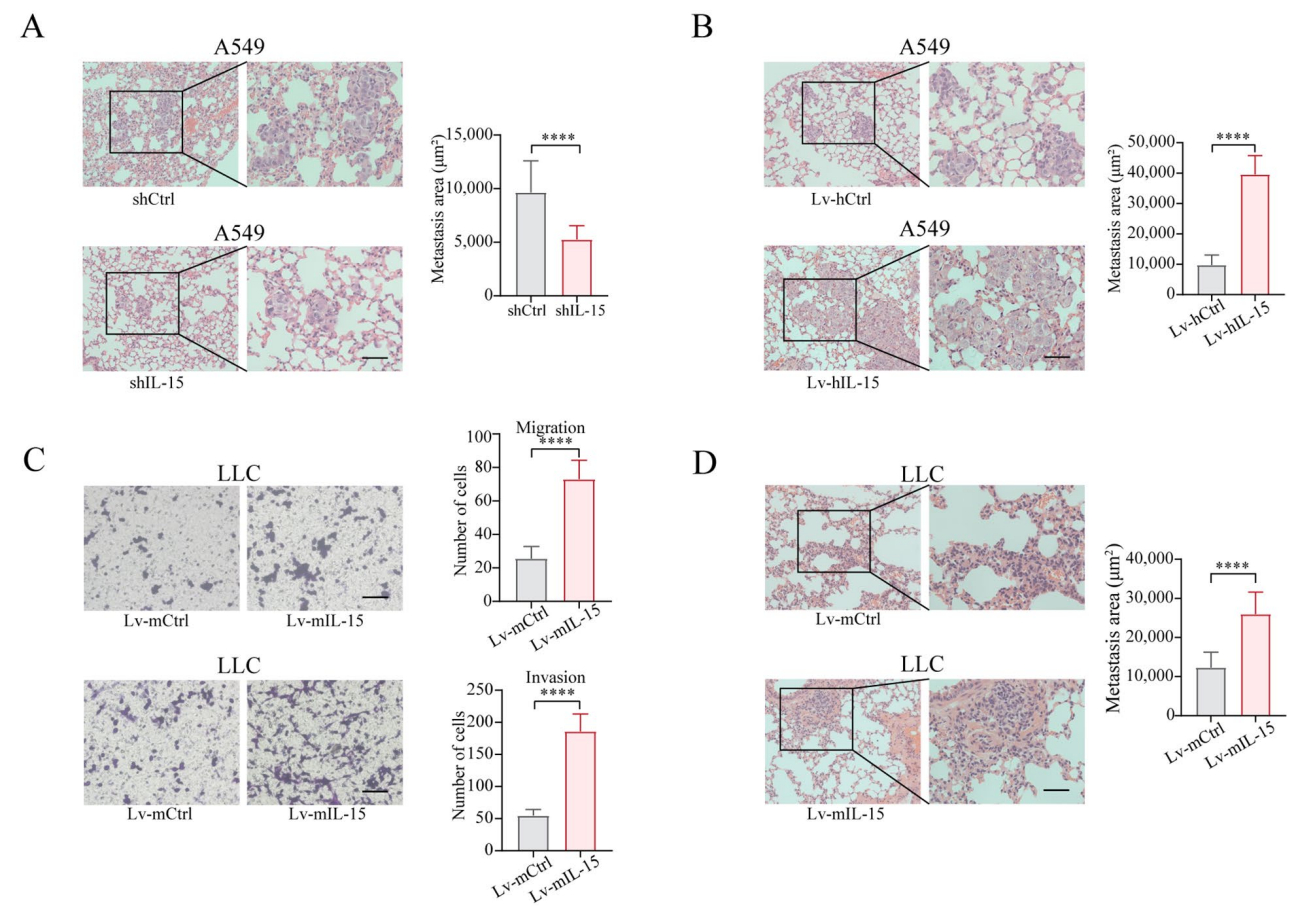
Cancer cell-intrinsic IL-15 promotes metastasis in vivo. (**A and B**), A549 cells transfected with shIL15 and shCtrl (**A**) or Lv-hIL-15 and Lv-hCtrl (**B**) were intravenously injected into NCG mice (1 × 10^6^ cells/mouse, *n* = 3). The mice were euthanized on day 7 after i.v. injection. Lung tissues were subjected to H&E staining. The area of lung metastasis was calculated. Scale bar, 50 μm. ****, *p* < 0.0001. (**C**), Transwell migration and invasion assays were performed on LLC cells transduced with Lv-mIL-15 and Lv-mCtrl. Scale bar, 100 μm. ****, *p* < 0.0001. (**D**), LLC cells transduced with Lv-mIL-15 or Lv-mCtrl were injected into C57BL/6 mice (1 × 10^6^ cells/mouse, *n* = 4) via the tail vein. The mice were euthanized on day 5 after i.v. injection. Lung tissues were subjected to H&E staining. Scale bar, 50 μm. ****, *p* < 0.0001. The area of lung metastasis was calculated as described in A and B. The data in the bar graphs are presented as the means ± SDs and were analyzed with a two-tailed Student’s *t* test

To determine whether endogenous IL-15 in tumor cells affects metastasis in vivo, we used well-established intravenous injection models of lung metastasis in mice. We generated an A549 cell line with stable genetic knockdown of IL-15 using shRNA (Figure S2A). The resultant shIL-15 A549 cells and shCtrl A549 cells were injected intravenously into immunodeficient NCG mice. Microscopic lung metastases were evaluated on day 7 after injection of the cells. A549 cells lacking IL-15 exhibited a significantly reduced ability to metastasize to the lung than cells transfected with shCtrl (Fig. 2A). Conversely, as shown in Figure S2B and Fig. 2B, IL-15-overexpressing A549 cells exhibited an increased capability for lung metastasis.

Next, using a murine LLC metastasis model [34–36], we evaluated the role of tumor cell-intrinsic IL-15 in regulating metastasis in immunocompetent mice. First, an IL-15-overexpressing LLC cell line (Lv-mIL-15) was generated (Figure S2C), and subsequently, the ability of these cells to migrate was assessed. Compared with Lv-mCtrl LLC cells, Lv-mIL-15 LLC cells exhibited increased migration and invasion (Fig. 2C). Like in previous results, LLC cells overexpressing IL-15 did not affect their proliferative capability compared to that of the control group (Figure S2D). To examine whether the overexpression of IL-15 in LLC cells facilitates metastasis formation in the lung, Lv-mIL-15 LLC cells were injected intravenously into C57BL/6 mice. Microscopic lung metastases were evaluated on days 3, 5, and 7 after injection of the cells. Overexpression of IL-15 in LLC cells facilitated the formation of metastases in the lung (Figure S2E and Fig. 2D). Thus, our data showed that cancer cell-intrinsic IL-15 promotes tumor metastasis in vivo.

### Cancer cell-intrinsic IL-15 increases filopodium formation through activation of the AKT-mTORC1-Cdc42 axis

**Fig. 3:**
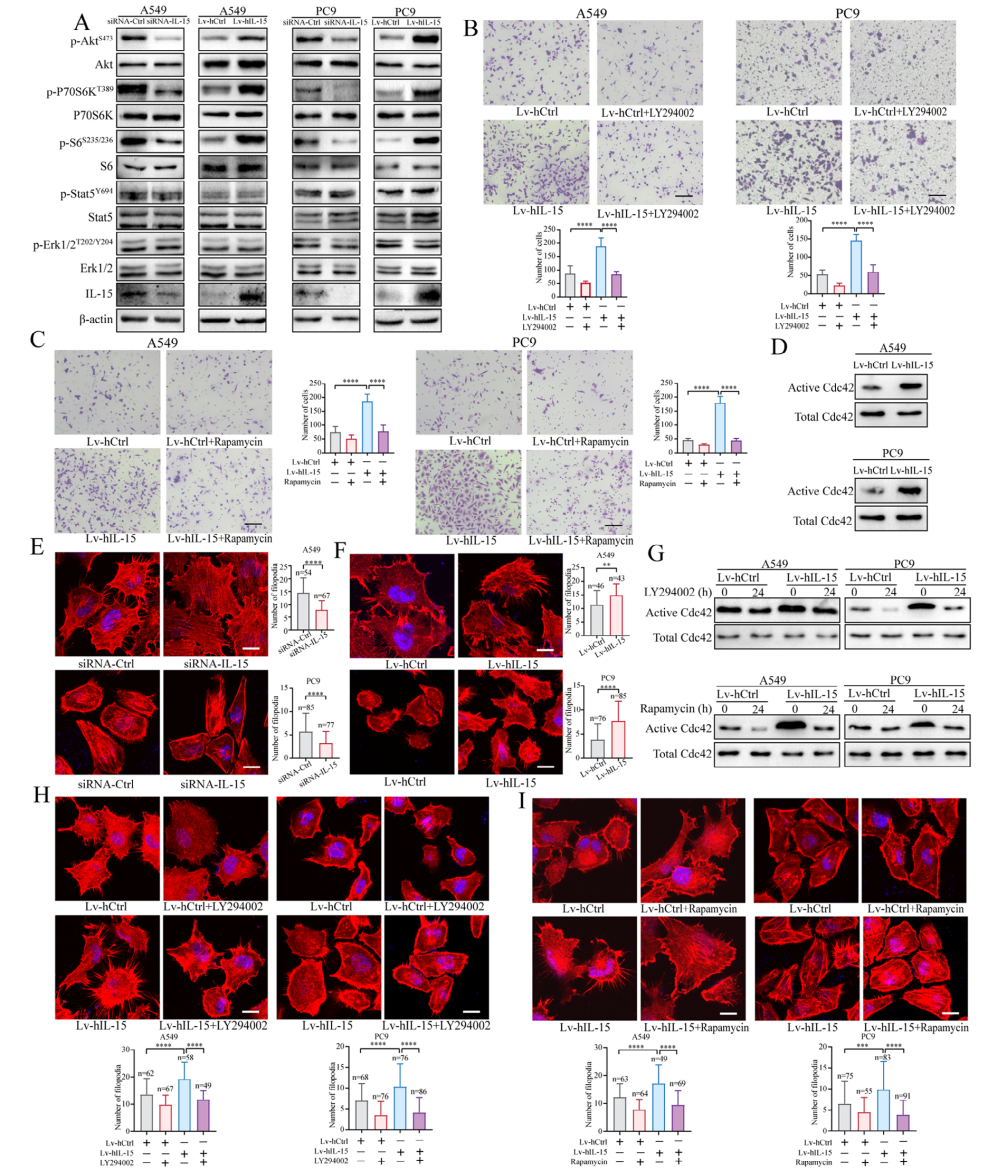
Cancer cell-intrinsic IL-15 activates the AKT-mTORC1-Cdc42 axis, leading to an increase in filopodium formation. (**A**), Phosphorylation of the indicated proteins was assessed by immunoblot analysis in A549 and PC9 cells transfected with siRNA-IL15 or siRNA-Ctrl or transduced with Lv-hIL-15 or Lv-hCtrl. (**B and C**), IL-15-overexpressing A549 and PC9 cells were treated with the PI3K inhibitor LY294002 (5 µM) (**B**) or the mTORC1 inhibitor rapamycin (500 nM) (**C**) for 24 h. Transwell migration assays were performed. Scale bar, 100 μm. The data in the bar graphs are presented as the means ± SDs and were analyzed with one-way ANOVA. ****, *p* < 0.0001. (**D**), A Cdc42 pulldown assay was performed on IL-15-overexpressing A549 and PC9 cells. (**E and F**), A549 and PC9 cells were transfected with siRNA-IL15 or siRNA-Ctrl for 48 h; subsequently, the cells were fixed and stained with Alexa Fluor 647-conjugated phalloidin to visualize F-actin (**E**). The same assay was performed on A549 and PC9 cells transduced with Lv-hIL-15 and Lv-hCtrl (**F**). The indicated number of cells (n) was used to quantify filopodia per cell. Two independent experiments were performed. Scale bar, 10 μm. **, *p* < 0.01; ****, *p* < 0.0001. (**G-I**), IL-15-overexpressing A549 and PC9 cells were cocultured separately with the inhibitors LY294002 (5 µM) and rapamycin (500 nM). The activity of Cdc42 was assessed by immunoblot analysis (**G**). The cells were fixed and stained with phalloidin (**H** and **I**). Scale bar, 10 μm. ***, *p* < 0.001; ****, *p* < 0.0001

To understand how intrinsic IL-15 affects the migration and invasion of cancer cells, we first investigated the signaling cascades mediated by intrinsic IL-15 in cancer cells. A lack of IL-15 expression in A549 and PC9 cells resulted in significantly reduced phosphorylation of AKT and inhibited the activation of mTORC1, as evidenced by reduced phosphorylation of p70S6K and S6, whereas overexpression of IL-15 in A549 and PC9 cells increased AKT phosphorylation and mTORC1 activity (Fig. 3A). Notably, modulation of IL-15 expression did not affect STAT5 or ERK phosphorylation. Next, to determine whether the AKT-mTORC1 pathway is involved in the IL-15-induced migration of tumor cells, we used the inhibitors LY294002 and rapamycin to inhibit AKT and mTORC1 activity, respectively. The migration of IL-15-overexpressing tumor cells was significantly compromised by the addition of LY294002 (Fig. 3B) or rapamycin (Fig. 3C).

A recent study showed that mTOR signaling regulates the morphology of outer radial glial cells by remodeling the cytoskeleton through the activity of the Rho-GTPase Cdc42 [37]. Thus, we first examined whether the overexpression of IL-15 alters Cdc42 activity in tumor cells. Cdc42 activity was assessed by measuring the amount of Cdc42 precipitated using a conformation-specific anti-Cdc42-GTP antibody. Compared with IL-15 wild-type A549 and PC9 cells, IL-15-overexpressing A549 and PC9 cells exhibited increased Cdc42 activity (Fig. 3D). Cdc42 reportedly controls the formation of actin bundle-containing filopodia at the cell periphery [38]. We next examined whether IL-15 induces morphological changes in tumor cells via cytoskeletal remodeling. Knockdown of IL-15 significantly reduced the number of filopodia in A549 and PC9 cells, whereas overexpression of IL-15 increased filopodium formation (Fig. 3E and F, respectively). Furthermore, the inhibition of AKT and mTORC1 in IL-15-overexpressing cells compromised Cdc42 activity (Fig. 3G) and filopodium formation (Fig. 3H and I). These data indicate that cancer cell-intrinsic IL-15 activates the AKT-mTORC1 pathway, leading to an increase in Cdc42 activity and remodeling of the cytoskeleton, which facilitates cell migration and invasion.

### Cancer cell-intrinsic IL-15 upregulates vimentin protein expression in an AKT-mTORC1-dependent manner

**Fig. 4:**
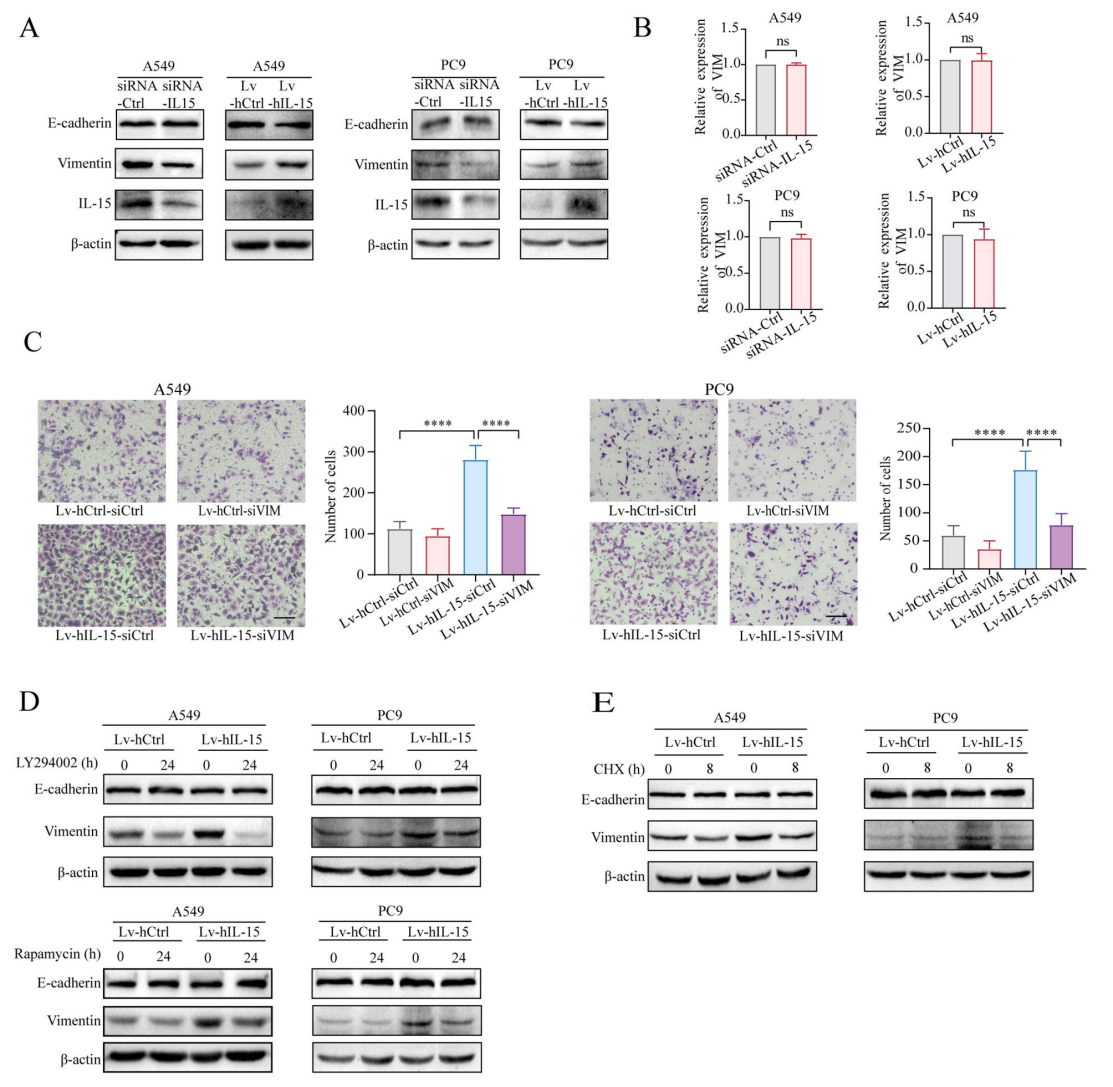
Cancer cell-intrinsic IL-15 upregulates vimentin protein expression in an AKT-mTORC1-dependent manner. (**A and B**), A549 and PC9 cells were transfected with siRNA-IL-15 and siRNA-Ctrl or transduced with Lv-hIL-15 and Lv-Ctrl. The cells were lysed and subjected to immunoblot analysis of E-cadherin and vimentin expression (**A**). RNA was extracted from the abovementioned cells and subsequently, RT-qPCR was performed to measure *VIM* expression (**B**). (**C**), IL-15-overexpressing A549 and PC9 cells were transfected with siRNA-*VIM* or siRNA-Ctrl for 48 h. Transwell migration assays were subsequently performed. Scale bar, 100 μm. ****, *p* < 0.0001. (**D**), IL-15-overexpressing A549 and PC9 cells were treated separately with the inhibitors LY294002 (5 µM) and rapamycin (500 nM) for 24 h. Then, vimentin expression was assessed via immunoblot analysis. (**E**), IL-15-overexpressing A549 and PC9 cells were treated with cycloheximide (50 µM) for 8 h and lysed for immunoblot analysis of E-cadherin and vimentin expression. Immunoblotting was also performed for β-actin as the protein loading control

As cancer cell-intrinsic IL-15 promotes cell migration and invasion, we examined whether IL-15 is involved in the process of EMT. Overexpression of IL-15 upregulated the expression of vimentin, a biomarker of mesenchymal-phenotype tumor cells, but did not affect the expression of E-cadherin, a biomarker of epithelial-phenotype tumor cells. Knockdown of IL-15 abrogated vimentin protein expression (Fig. 4A). The levels of EMT-associated transcription factors such as Snail, Slug, and ZEB1, were not significantly affected by alterations in IL-15 expression levels (Figure S3). Interestingly, overexpression of IL-15 did not affect *VIM* mRNA expression (Fig. 4B). Vimentin plays a crucial role in the acquisition of migratory and invasive capabilities by tumor cells [39, 40]. As shown in Fig. 4C, knocking down vimentin in Lv-hIL-15 A549 cells and PC9 cells reversed the IL-15-mediated increase in migration.

To determine whether IL-15 overexpression-mediated activation of the AKT-mTORC1 pathway is responsible for the upregulation of vimentin expression, we used LY294002 and rapamycin to inhibit AKT and mTORC1 activity, respectively, in cultured IL-15-overexpressing tumor cells. Inhibition of AKT and mTORC1 abrogated IL-15-induced vimentin expression, as determined by immunoblot analysis (Fig. 4D). We speculated that the regulatory effect of the AKT-mTORC1 pathway on protein translation may contribute to the IL-15-mediated upregulation of vimentin expression. The addition of cycloheximide (CHX), a widely used translation inhibitor, to the cell culture reversed the upregulation of vimentin expression in the IL-15-overexpressing tumor cells (Fig. 4E). Thus, these results indicate that in addition to activating Cdc42 and promoting filopodium formation, AKT-mTORC1 pathway activity also induces partial EMT, as evidenced by increased vimentin expression.

### A high level of IL-15 expression in lung adenocarcinoma cells is related to a favorable prognosis

**Fig. 5:**
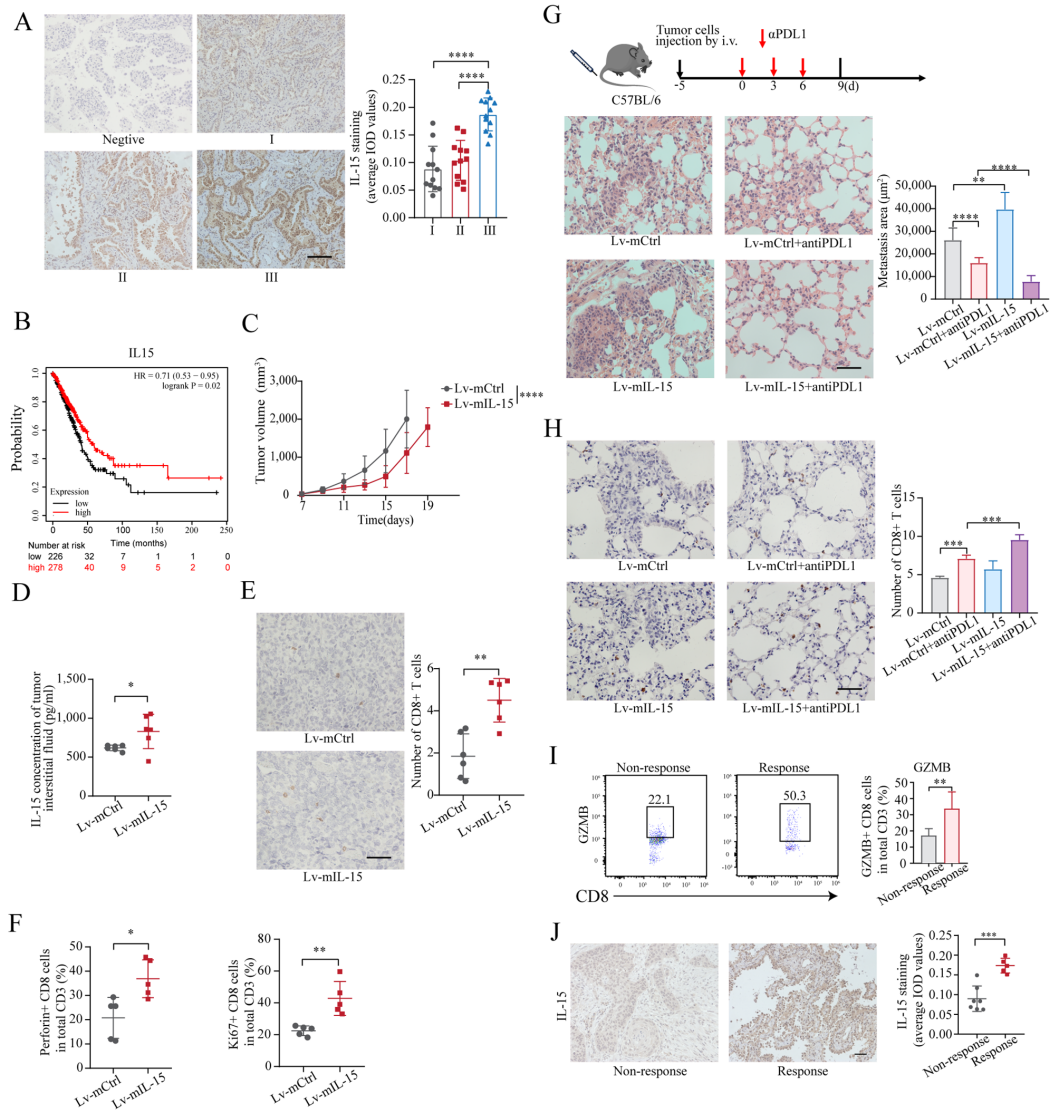
A high level of IL-15 expression in lung adenocarcinoma is related to a favorable prognosis. (**A**), Expression of IL-15 in lung adenocarcinoma cells at different stages (*n* = 12 samples per stage). Scale bar, 100 μm. ****, *p* < 0.0001. (**B**), Kaplan-Meier plotter analysis of survival probability according to the expression level of IL-15 (high expression, *n* = 278; low expression, *n* = 226, *p* = 0.0202). (**C**), LLC cells transduced with Lv-mIL-15 or Lv-mCtrl were inoculated subcutaneously into C57BL/6 mice (1 × 10^6^ cells/mouse, *n* = 7 mice/group). Tumor volumes were calculated, and the data are presented as the means ± SDs. Two-way ANOVA followed by Sidak’s multiple comparison test was used. ****, *p* < 0.0001. (**D**), The concentration of IL-15 in the tumor interstitial fluid was measured via ELISA. Each point in the graph represents an individual mouse. The data are presented as the means ± SDs. *, *p* < 0.05. (**E**), Representative images of tumor-infiltrating CD8^+^ T cells. Scale bar, 50 μm. **, *p* < 0.01. (**F**), TILs were enriched in the tumors and stained with antibodies against CD45, CD3, CD8, Ki67, and perforin. The data are presented as the means ± SDs (*n* = 5). *, *p* < 0.05; **, *p* < 0.01. (**G**), LLC cells transduced with Lv-mIL-15 (*n* = 5) or Lv-mCtrl (*n* = 3) were injected intravenously into C57BL/6 mice. The mice were treated with an isotype control or an anti-PD-L1 antibody (10 mg/kg). The mice were euthanized on day 9 after the initial anti-PD-L1 treatment. Lung tissues were subjected to H&E staining. Scale bar, 50 μm. **, *p* < 0.01; ****, *p* < 0.0001. (**H**), Representative images of tumor-infiltrating CD8^+^ T cells in the lungs of C57BL/6 mice. Scale bar, 50 μm. ***, *p* < 0.001. (**I**), Twelve freshly collected human lung tumor explants were treated with the anti-PD-1 mAb (10 µg/ml) for 24 h. TILs were isolated from these explants and stained with antibodies against granzyme B. Five explants responded to anti-PD-1 treatment, while seven explants did not respond to anti-PD-1 treatment. **, *p* < 0.01. (**J**), The levels of IL-15 expression in human lung tumor samples were evaluated using the average integrated optical density (average IOD). ***, *p* < 0.001

Having demonstrated that cancer cell-intrinsic IL-15 promotes the aggressiveness of cancer cells (Figs. 1, 2, 3 and 4), we next examined whether tumor cell-specific IL-15 is associated with the progression of lung adenocarcinoma. As shown in Fig. 5A, a high expression level of IL-15 in tumor cells was associated with a high TNM stage. We thus speculated that a high expression level of IL-15 might be associated with poor survival. Using the online survival analysis tool Kaplan-Meier Plotter (http://kmplot.com/analysis), we analyzed the association between IL-15 expression and survival probability in patients with lung adenocarcinoma (Figure 5B). The cohort was divided into two groups according to the median expression level (IL-15^high^ group, *n* = 278; IL-15^low^ group, *n* = 226). Unexpectedly, a high level of IL-15 in tumors was associated with an increased survival probability. To understand these seemingly inconsistent conclusions, we utilized a mouse model to examine the role of cancer cell-intrinsic IL-15 in tumor growth in vivo. C57BL/6 mice were subcutaneously implanted with Lv-mIL-15 LLC cells or Lv-mCtrl LLC cells. The growth of tumors derived from implanted Lv-mIL-15 LLC cells was slower than that of tumors derived from implanted Lv-mCtrl LLC cells (Fig. 5C, Figure S4A and S4B).

IL-15 plays a pivotal role in enhancing antitumor immune responses mediated by T lymphocytes. We thus measured the concentration of intercellular IL-15 in wild-type LLC tumors and IL-15-overexpressing LLC tumors. The concentration of intercellular IL-15 was significantly greater in tumors derived from LLC cells transduced with Lv-mIL-15 than in the corresponding control LLC tumors (Fig. 5D). Notably, tumor necrosis, which is characterized by shrunken nuclear fragments and clusters of dead and degraded tumor cells, was observed (Figure S4C) [41], indicating that intracellular IL-15 can be released from dead Lv-mIL-15 LLC cells. Consistent with the increase in intercellular IL-15, the density of tumor-infiltrating CD8^+^ T lymphocytes was greater in Lv-mIL-15-transfected LLC tumors than in control LLC tumors (Fig. 5E); moreover, the effector functions of these tumor-infiltrating CD8^+^ T cells were also enhanced, as evidenced by the increased proportion of perforin-positive and Ki-67-positive T cells compared to that in control LLC tumors (Fig. 5F). Our data indicate that the highly expressed IL-15 in tumor cells may participate in the antitumor immune response in vivo.

Next, we sought to determine whether IL-15-overexpressing LLC cells are more sensitive to anti-PD-L1 therapy. C57BL/6 mice were subcutaneously implanted with Lv-mIL-15 LLC cells or Lv-mCtrl LLC cells and treated with an anti-PD-L1 antibody (day 0). Tumor growth induced by the implantion Lv-mIL-15 LLC cells was slower than that induced by the implantation of Lv-mCtrl LLC cells (Figure S4D). The intercellular level of IL-15 and density of tumor-infiltrating CD8 + T lymphocytes were greater in Lv-mIL-15-transfected LLC tumors than in control LLC tumors (Figure S4E and S4F). The proportions of perforin-positive and Ki-67-positive T cells were greater than those in control LLC tumors (Figure S4G). In addition, we used a mouse model of lung metastasis as previously described (Figure S2E and Fig. 2D). C57BL/6 mice were intravenously injected with Lv-mIL-15 LLC cells or Lv-mCtrl LLC cells. After 5 days, the two groups of mice were randomly separated into two subgroups. The mice in one subgroup were treated with an anti-PD-L1 antibody (day 0), and the mice in the other subgroup were treated with an isotype control. As shown in Fig. 5G, anti-PD-L1 therapy reduced LLC metastasis, and more importantly, anti-PD-L1 therapy almost completely abolished the formation of metastases derived from IL-15-overexpressing LLC cells. The infiltration of CD8^+^ T cells into lung tissues was significantly increased in LLC tumor-bearing mice after treatment with anti-PD-L1 antibodies, and even more CD8^+^ T cells infiltrated the lung tissue in Lv-mIL-15 LLC tumors after anti-PD-L1 therapy (Fig. 5H). Additionally, we used a human lung cancer explant model to verify the in vivo findings in an animal model. As shown in Figure S4H, for the tumor explants treated with the anti-PD-1 mAb, five out of the 12 tumor explants responded, as evidenced by an increased percentage of granzyme B-positive CD8 + T cells. Interestingly, the level of IL-15 expression in tumor cells was significantly greater in the responding tumors than in the nonresponsive tumors (Fig. 5I and J). Our data suggest that although cancer cell-intrinsic IL-15 can promote metastasis, high expression of IL-15 in tumor cells increases the amount of IL-15 in the tumor microenvironment and enhances the efficacy of anti-PD-L1 immunotherapy.

### Exogenous Il-15 inhibits cancer cell motility and migration

**Fig. 6:**
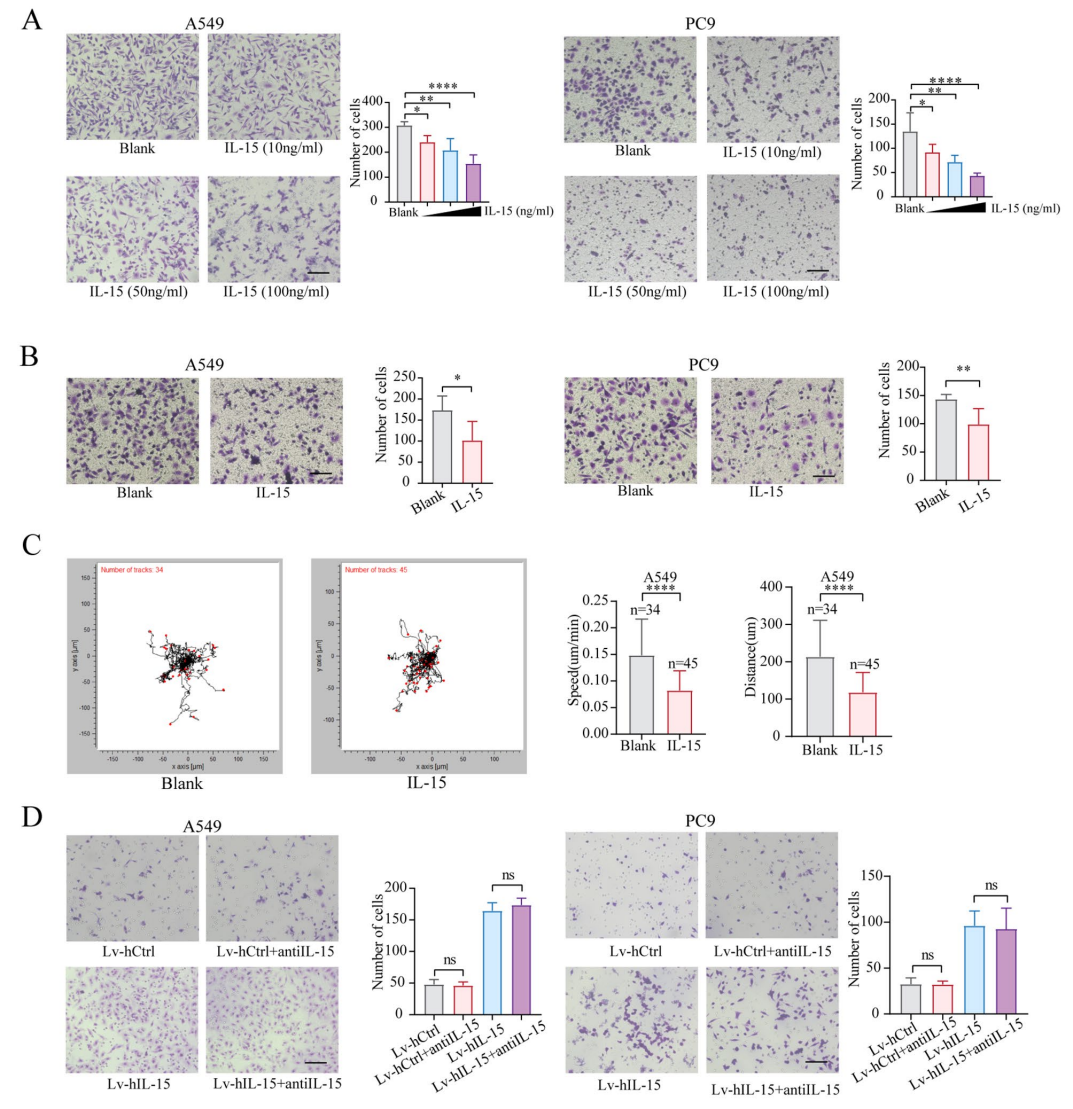
Exogenous IL15 inhibits tumor cell migration and invasion. (**A and B**), Transwell migration (**A**) and invasion (**B**) assays were performed on A549 and PC9 cells in the presence or absence of rIL15. The data are presented as the means ± SDs and were analyzed with one-way ANOVA. Scale bar, 100 μm. *, *p* < 0.05; **, *p* < 0.01; ****, *p* < 0.0001. (**C**), A549 cells were cultured with rIL15 (100 ng/mL). The motion trajectory of the A549 cells was recorded using a live-cell analysis system for 24 h. The distance and speed data are presented as the means ± SDs and were analyzed with two-tailed Student’s *t* test. ****, *p* < 0.0001. (**D**), A Transwell migration assay was performed on IL-15-overexpressing A549 and PC9 cells in the presence or absence of an anti-IL15 neutralizing antibody (10 µg/mL). Scale bar, 100 μm

As shown in Fig. 5D, intercellular IL-15 levels were increased in Lv-mIL-15 LLC tumors in vivo. We then sought to determine whether extracellular IL-15 affects cell motility and migration. The addition of exogenous IL-15 to the cell cultures did not affect the proliferation of A549 or PC9 cells (Figure S5A). Interestingly, in contrast to cancer cell-intrinsic IL-15, exogenous IL-15 inhibited cell migration in a dose-dependent manner (Fig. 6A). This extracellular IL-15 also inhibited tumor cell invasion and motility (Fig. 6B and C). Notably, extracellular IL-15 did not affect E-cadherin or vimentin expression (Figure S5B).

IL-15 has two known isoforms containing either a long signal peptide (LSP) or a short signal peptide (SSP), and these isoforms are produced from alternatively spliced transcripts. It has been proposed that the SSP-containing isoform of IL-15 remains exclusively intracellular and that LSP IL-15 can be secreted [42, 43]. As shown in Figure S5C, both the SSP isoform-encoding and LSP isoform-encoding transcripts were expressed in tumors and lung adenocarcinoma cell lines. As shown in Figure S5D, the amount of IL-15 obtained from Lv-hIL-15-transfected A549 or control A549 cells was comparable; however, the levels of IL-15 in the cell lysates obtained from Lv-hIL-15 A549 cells were significantly greater than those in Lv-hCtrl A549 cells. Similar findings were observed in LLC cells transfected with Lv-mIl-15. These data suggest that IL-15 expressed by tumor cells is mainly retained intracellularly. To further determine whether the effect of cell-intrinsic IL-15 on cell migration is mediated by intracellular IL-15 rather than by extracellular IL-15, an anti-IL-15 neutralizing antibody was added to cultured Lv-hIL-15 A549 and PC9 cells, after which cell migration was assessed. We first assessed the efficacy of the IL-15 neutralizing antibody. As shown in Figure S5E, the IL-15 neutralizing antibody effectively inhibited the IL-15-mediated activation of T cells, as evidenced by Ki-67 expression. Neutralizing extracellular IL-15 did not change the migration of Lv-hIL-15 cells compared to that of their counterparts (Fig. 6D), suggesting that intracellular IL-15 but not extracellular IL-15 contributes to the promotive effect of cancer cell-intrinsic IL-15 on cell migration.

### Exogenous IL-15 inhibits stress fiber and focal adhesion formation by downregulating the activity of the RhoA-myosin light chain (MLC2) axis

**Fig. 7:**
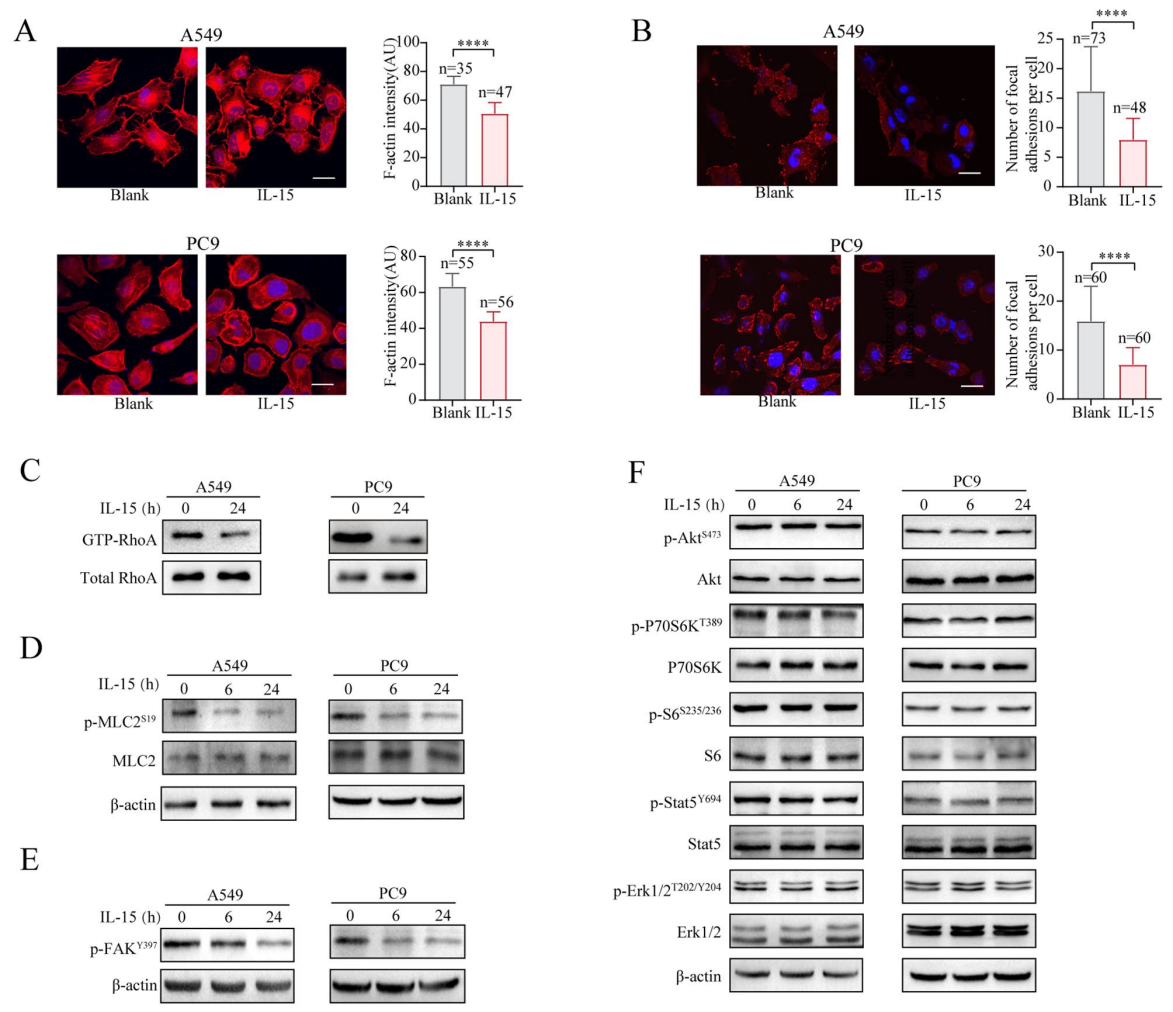
Exogenous IL15 inhibits stress fiber and focal adhesion formation via downregulation of RhoA activity. (**A-E**), A549 and PC9 cells were treated with rIL-15 (100 ng/mL). The formation of actin stress fibers (**A**) and vinculin expression (**B**) were assessed. Scale bar, 20 μm. ****, *p* < 0.0001. A pulldown assay was performed to detect the active form of RhoA (**C**). Phosphorylated MLC2 (**D**) and FAK (**E**) were analyzed by immunoblotting. (**F**), A549 and PC9 cells were treated with rIL-15 (100 ng/mL) for 6–24 h. The expression levels of the indicated proteins were measured by immunoblotting

Unlike IL-15 overexpression within tumor cells, exogenous IL-15 did not affect the formation of pseudopodia in A549 cells or PC9 cells; however, compared with that in untreated cells, the number of stress fibers in tumor cells was significantly lower (Fig. 7A). The number of focal adhesions in IL-15-treated cells was also dramatically reduced (Fig. 7B). These exogenous IL-15-induced morphological changes are consistent with the function of RhoA [44, 45]. Thus, we examined whether exogenous IL-15 affects RhoA activity. RhoA, a member of the Rho family of small GTPases, is a key regulator of the actin cytoskeleton and is particularly related to the changes in cell shape and adhesion dynamics that drive cell migration [46]. The addition of IL-15 to the cell cultures resulted in a decrease in the abundance of GTP-bound RhoA (Fig. 7C). One of the downstream effectors of RhoA is MLC2, which plays a central role in regulating cell motility [47]. Phosphorylation of MLC2 at serine 19 promotes actin-myosin cross-bridging, which leads to cell contraction [48]. Exogenous IL15 significantly downregulated the phosphorylation of MLC2 (Fig. 7D). RhoA also activates focal adhesion kinase (FAK) signaling [49, 50], and additional IL-15 significantly reduced the phosphorylation of FAK (Fig. 7E). Notably, exogenous IL-15 did not affect the status of the AKT-mTORC1 or STAT5 pathways in tumor cells (Fig. 7F).

### Cancer cell-intrinsic and extracellular IL-15 differentially regulate cell morphology and migration

**Fig. 8:**
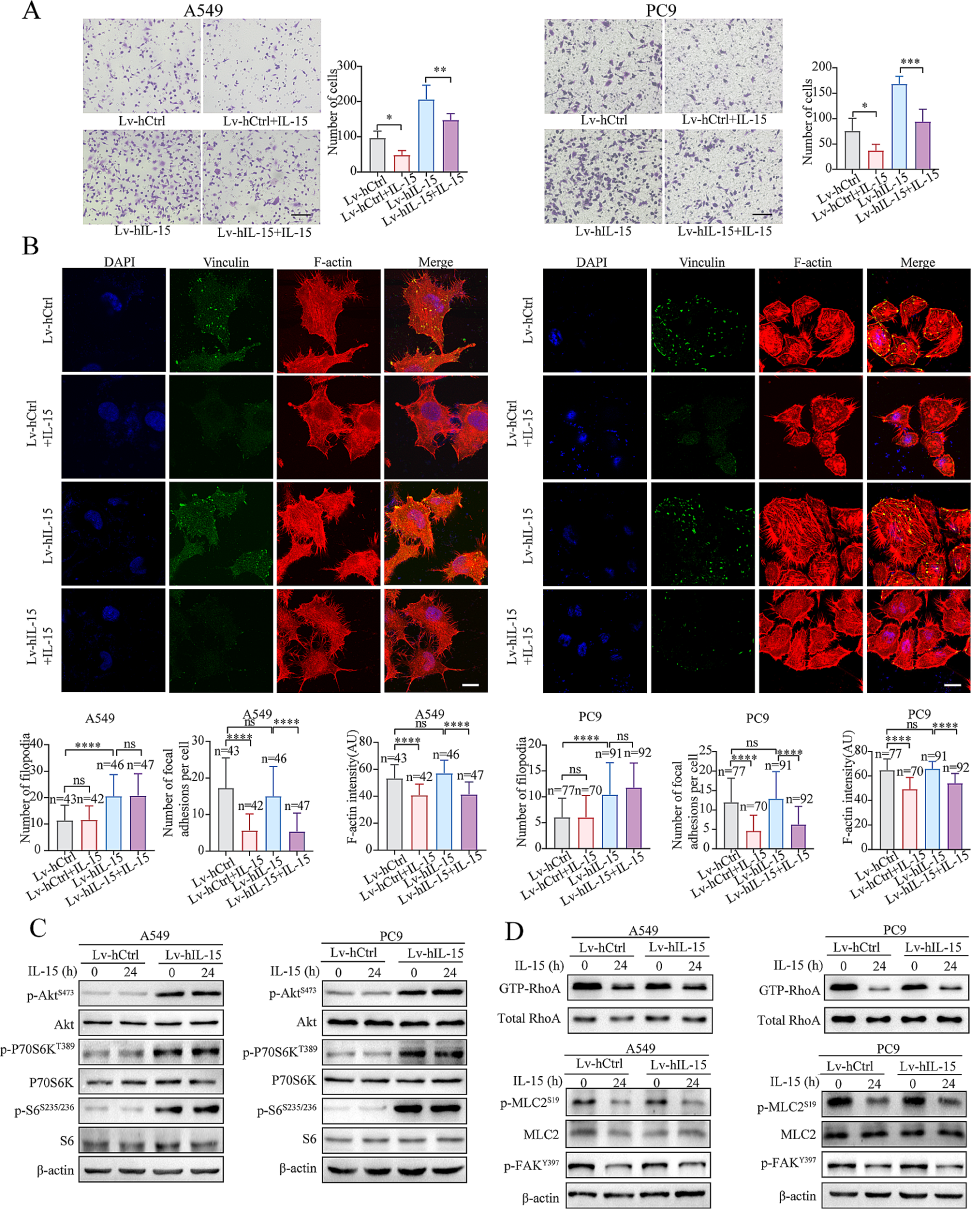
Exogenous and intracellular IL15 differentially regulate IL-15-mediated signaling, cell morphological changes, and migration. **A**, A Transwell migration assay was performed on IL-15-overexpressing A549 and PC9 cells treated with exogenous IL-15 (100 ng/mL) for 24 h. Scale bar, 100 μm.*, *p* < 0.05; **, *p *< 0.01; ***, *p* < 0.001. **B-D,** IL-15-overexpressing A549 and PC9 cells were treated with rIL-15 for 24 h. Then, cell morphological changes were evaluated by confocal fluorescence microscopy (B).Scale bar, 10 μm.****, *p* < 0.0001. The expression levels of the indicated proteins were measured by immunoblotting (C). Cell lysates were subjected to a pulldown assay to detect GTP-RhoA and the indicated proteins (D)

As intracellular and extracellular IL-15 clearly differentially regulate cell migration, we investigated how the migration of tumor cells is regulated by both cell-intrinsic IL15 and extracellular IL-15. As shown in Fig. 8A, exogenous IL-15 significantly reduced the migration of Lv-hIL-15 A549 and PC9 cells. Moreover, although exogenous IL-15 did not affect intrinsic IL-15-induced filopodium formation, it reduced the formation of adhesion plaques (Fig. 8B). Exogenous IL-15 did not affect Cdc42 activity (Figure S5F). Consistent with these findings, exogenous IL-15 did not affect cancer cell-intrinsic IL-15-mediated activation of the AKT-mTORC1pathway (Fig. 8C), but its effect on reducing RhoA activity and the phosphorylation of MLC2 and FAK was not affected (Fig. 8D). Conversely, overexpression of IL-15 did not affect the activity of RhoA or the phosphorylation of MLC2 or FAK. The PI3K inhibitor LY294002 abrogated the intracellular IL-15-mediated increase in cell migration (Fig. 3B); however, LY294002 did not affect the phosphorylation of MLC2 or FAK (Figure S5G). These results suggest that cancer cell-intrinsic IL-15 and exogenous IL-15 utilize different signaling pathways and downstream events to regulate the morphology, motility, and migration of tumor cells.

### IL-15Rα expressed by tumor cells is needed for the actions of intracellular and extracellular IL15 on tumor cells

**Fig. 9:**
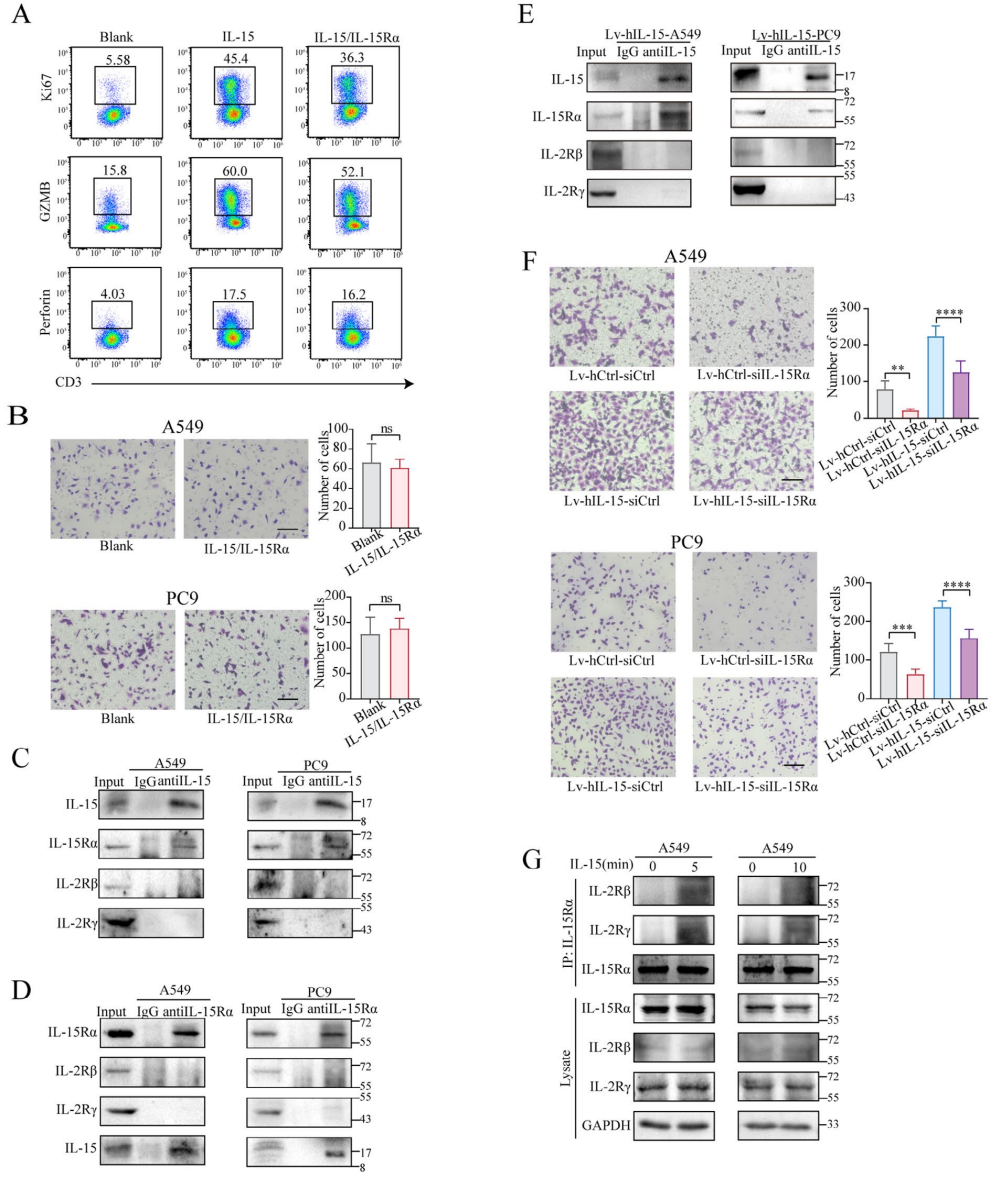
IL-15Rα is differentially associated with the IL-15 receptor β/ γ upon engaging extracellular and intracellular IL-15. (**A**), CD3^+^ T cells isolated from PBMCs were stimulated with the IL15/IL15Rα complex (100 ng/mL) for 3 days. The expression of Ki67, GZMB, and perforin was assessed by flow cytometry. (**B**), A Transwell migration assay was performed on A549 and PC9 cells in the presence or absence of the IL15/IL15Rα complex (100 ng/mL). The data are presented as the means ± SDs. Scale bar, 100 μm. (**C and D**), Antibodies against IL15 (**C**) or IL15Rα (**D**) were added to the lysates of A549 and PC9 cells; (**E**), Antibodies against IL15 were added to the lysates of IL-15- overexpressing A549 and PC9 cells. The proteins pulled down by the antibodies were analyzed by immunoblotting. The protein levels of IL15Rα, IL2/IL-15Rβ, and IL2/IL-15Rγ were measured. (**F**), Transwell migration assays were performed on IL-15-overexpressing A549 and PC9 cells that were transfected with siRNA-IL15Rα or siRNA-Ctrl for 48 h. The data are presented as the means ± SDs and were analyzed via one-way ANOVA. Scale bar, 100 μm.**, *p* < 0.01; ***, *P* < 0.001; ****, *p* < 0.0001. (**G**), Antibodies against IL15Rα were added to the lysates of A549 cells treated with or without IL-15 for 5 or 10 min after which the proteins pulled down by the antibodies were analyzed by immunoblotting. The protein levels of IL15Rα, IL2/IL-15Rβ, and IL2/IL-15Rγ were measured

Several studies have reported that IL-15 requires the cell membrane IL-15Rα to perform its function [51–54]. We thus examined whether the IL-15-IL-15Ra complex can affect tumor cell migration. As shown in Fig. 9A, the complex significantly enhanced the effector function of CD3^+^ T cells, as evidenced by the upregulation of Ki-67, granzyme B (GZMB), and perforin expression. However, the complex did not affect tumor cell migration (Fig. 9B). These results strongly suggest that the action of exogenous IL-15 on tumor cells requires cell membrane IL-15Rα.

To determine whether intracellular IL-15 also requires IL-15Rα to function, we used anti-IL-15 antibodies to immunoprecipitate cancer cell-intrinsic IL-15 and found that IL-15 was associated with IL-15Rα (Fig. 9C and D) but not with the IL-2/IL-15Rβ or IL-2Rγ chains. In IL-15-overexpressing A549 cells, IL-2/IL-15Rβ and IL-2/IL-15Rγ were still not detected in the complex formed by intracellular IL-15 and IL-15Rα (Fig. 9E). Knockdown of IL-15Rα in IL-15-overexpressing A549 and PC9 cells led to a significant reduction in cell migration (Fig. 9F), suggesting that the promotion of cell migration by intrinsic IL-15 also requires IL-15Rα. As shown in Figure S6A, knockdown of IL-15Rα decreased the phosphorylation levels of AKT, p70S6K, and S6. Furthermore, the activity of Cdc42 was reduced after knocking down IL-15Rα (Figure S6B). Moreover, the lack of IL-15Rα led to reduction in the number of filopodia in A549 and PC9 cells (Figure S6C). These results verify the importance of IL-15Rα in mediating the migration of cells via intracellular IL-15.

We next sought to determine whether exogenous IL-15 engages the IL-15 receptor via a distinct binding mode. To this end, we used antibodies against IL-15Rα to perform immunoprecipitation assays. As shown in Fig. 9G, upon engagement of tumor cells by exogenous IL-15, we detected the association of IL-15Rα with the IL-2/IL-15Rβ and IL-2Rγ chains. Our results suggest that the differential actions of intracellular and extracellular IL-15 on tumor cells might be caused by their distinctive modes of engaging the IL-15 receptor.

## Discussion

Our study demonstrated for the first time that tumor cell-intrinsic IL-15 contributes to cancer cell aggressiveness, as evidenced by the promotion of cell migration and invasion. Intracellular IL-15 increased the activity of Cdc42 through activation of the AKT-mTORC1 pathway, leading to an alteration in cell morphology and increased filopodium formation. Activation of the AKT-mTORC1 pathway also led to upregulation of vimentin protein expression, which contributed to the promotion of cell migration and invasion. Interestingly, exogenous IL-15 decreased cell motility and migration, consistent with the findings of other reports [19, 55]. Exogenous IL-15 did not affect the AKT-mTORC1-Cdc42 pathway but instead reduced RhoA activity and FAK phosphorylation, resulting in decreased formation of stress fibers and focal adhesions and inhibition of cell migration and invasion. These results indicate that the signaling pathway activated through intracellular IL-15 differs from that mediated by extracellular IL-15 and regulates various processes related to cell motility and migration. Biological structures, such as filopodia, focal adhesions, and stress fibers are involved in cell movement. The relationship between filopodia and focal adhesions is tightly coordinated and crucial for cell movement [56]. However, the underlying mechanisms regulating focal adhesion and filopodium formation are independent [56–58]. Several studies have shown that focal adhesions and stress fibers are controlled by RhoA, while the formation of filopodia depends on Cdc42 [57]. Knockdown of vinculin, which is crucial for focal adhesions, does not affect the number of cellular filopodia [58].

The differential effects of intracellular and extracellular IL-15 on the cellular behaviors of tumor cells might be related to their mechanisms of action. Like other cytokines, IL-15 can activate the trimeric receptor IL-15Rα/β/γ, which is expressed by target cells (cis-presentation). IL-15Rα can also trans-present IL-15 to target cells expressing IL-2/IL-15Rβ/γ. The short cytoplasmic tails of IL-15Rα in the presence of IL-2/IL-15Rβ and IL-2/IL-15Rγ do not seem to participate in signal transduction [59]. However, Pereno et al. reported that in human melanoma cells, intracellular IL-15/IL-15Rα binds to the transducing molecule TRAF2 and subsequently activates NF-κB, suggesting that the intracellular IL-15/IL-15Rα complex enables signal transduction through the IL-15Rα-associated molecules [60]. A study using a T-cell line expressing IL-15Rα/β/γ or IL-15Rβ/γ showed that IL-15 cis- and trans-presentation lead to different dynamics of receptor activation and signal transduction [61]. Both trans- and cis-presentation enable the induction of immune responses mediated by NK and T cells and activate the JAK-STAT5 and PI3K-AKT pathways [62, 63]. Several findings show that trans-presentation is a major mechanism mediating IL-15 responses [64]. However, the mechanisms by which the different modes of action of IL-15 affect tumor cells have not been fully investigated. Our data showed that exogenous IL-15, but not the IL-15-IL-15Rα complex inhibited cell migration, suggesting that the action of exogenous IL-15 requires the presence of tumor cell IL-15Rα and cis-presentation. We also found that tumor cell IL-15Rα was associated with IL-15β and IL-15Rγ upon engaging with extracellular IL-15. Intracellular IL-15 was shown to be associated with only IL-15Rα and not with IL-15Rβ/γ. The mechanism underlying how the intracellular IL-15/IL-15Rα complex transduces signaling has not been elucidated, and further investigation is warranted.

Both known isoforms of human IL-15 contain either an LSP or an SSP. The SSP regulates the fate of the mature IL-15 protein by controlling its intracellular trafficking to nonendoplasmic reticulum sites, whereas the LSP regulates the rate of protein translation and functions as a secretory signal peptide. The IL-15 isoform with the SSP is exclusively stored intracellularly, and is not typical of soluble interleukin systems [65]. When SSP and LSP are coexpressed in the same cell in the presence of IL-15Rα, they compete for binding to IL-15Rα. The interaction of SSP IL-15 with IL-15Rα leads to the formation of a complex with decreased stability, and SSP IL-15 functions as a competitive inhibitor of LSP IL-15 [43]. Our results showed that both isoforms of IL-15 were transcribed in lung adenocarcinoma cells, suggesting that the IL-15 produced in tumor cells could be either secreted or stored intracellularly. Notably, in this study, we did not detect IL-15 in the tumor cell culture supernatants by classical ELISA and it was not increased even in the supernatants collected from tumor cells transfected with IL-15. Interestingly, we inoculated IL-15-overexpressing LLC cells and control LLC cells subcutaneously, measured the concentration of intercellular IL-15 in tumors by ELISA and found a significant increase in IL-15 in LLC tumors derived from IL-15-overexpressing cells compared to that in control LLC tumors. An increased amount of IL-15 in the tumor interstitial fluid could be released predominantly from dead tumor cells, as we observed necrosis of LLC cells in the subcutaneous tumors. Several studies have reported that the secretion of IL-15 by live cells is challenging due to complex transcriptional and translational regulation of IL-15 [65, 66]. The presence of AUGs in the 5’-untranslated region (UTR) of IL-15 interferes with IL-15 mRNA translation [67]. The nucleotide or protein sequences of the IL-15 signal peptide and carboxyl terminus also contribute to poor translation and secretion of IL-15 transcripts [42, 68]. Because of the presence of these regulatory elements, the amount of secreted IL-15 may not significantly increase [69].

IL-15 has been proposed to function as a danger molecule that signals to the immune system that a tissue is under attack and primes the immune system to mediate tissue destruction [70]. Intracellular IL-15 in tumors is crucial for the induction of optimal antitumor responses. Indeed, our study showed that the overexpression of IL-15 in tumor cells facilitated antitumor immune responses, as evidenced by an increase in the number and enhancement of effector functions of CD8^+^ T cells in tumors compared to those in wild-type LLC tumors. Our results were consistent with the findings reported by Araki et al., who showed that weakly immunogenic colon 26 cells transfected with murine mature-IL-15 cDNA, which secreted high levels of IL-15, were rejected completely upon injection into syngeneic BALB/c mice via the actions of CD8^+^ T cells [71].

IL-15 and the IL-15/IL-15Rα complex have been evaluated for antitumor efficacy in preclinical studies and clinical trials. The IL-15/IL-15Rα complex has a longer half-life than the other complex and is more sensitive to immune cells; thus, its design and modification have received increased amounts of attention [72, 73]. Although the IL-15/IL-15Rα complex significantly improves the cytolytic function of NK cells and CD8^+^ T cells, upon treatment with the IL-15/IL-15Rα agonist N-803, this hyperresponsiveness of NK cells is detrimental, leading to subsequent NK cell tolerance and poor lasting antitumor efficacy [74, 75]. The failure of the IL-15/IL-15Rα complex in the treatment of cancer has not been considered to date in light of its actions on tumor cells. Our data showed that, unlike monomeric IL-15, the IL-15/IL-15Rα complex cannot antagonize the cancer cell-intrinsic IL-15-mediated promotion of cancer cell aggressiveness. Thus, the impact of IL-15 on tumor cells should not be overlooked when focusing on its impact on immune cells to more effectively apply IL-15 in cancer therapy.

Additionally, using an LLC lung metastasis model, we showed that tumor cells transfected with IL-15 exhibited dramatically increased sensitivity to anti-PD-L1 therapy, although the overexpression of IL-15 in tumor cells potentiated their ability to metastasize to the lung. Our results demonstrated for the first time that IL-15 expressed by cancer cells functions as a double-edged sword in tumor progression. On the one hand, intracellular IL-15 promotes tumor cell migration and metastasis in vivo; on the other hand, extracellular IL-15 suppresses tumor progression by inhibiting tumor cell migration and enhancing antitumor immune responses. Thus, high levels of IL-15 expressed by tumor cells improve the responsiveness of tumors to PD-1/PD-L1 blockade-based immunotherapies.

### Electronic supplementary material

Below is the link to the electronic supplementary material.

Supplementary Material 1

Supplementary Material 2

Supplementary Material 3

Supplementary Material 4

Supplementary Material 5

Supplementary Material 6

Supplementary Material 7

Supplementary Material 8

Supplementary Material 9

## Data Availability

No datasets were generated or analysed during the current study.

## Abbreviations

ANOVA: Analysis of variance
CHX: Cycloheximide
DAB: Diaminobenzidine
ECL: Enhanced chemiluminescence
ELISA: Enzyme linked immunosorbent assay
EMT: Epithelial-mesenchymal transition
FAK: Focal adhesion kinase
FBS: Fetal bovine serum
GZMB: Granzyme B
HE: Hematoxylin and eosin
HRP: Horseradish peroxidase
ICB: Immune checkpoint blockade
IL-15: Interleukin-15
IL15Rα: Interleukin-15 receptor alpha subunit
IL-2Rβ: Interleukin-2 receptor β subunit
IL-2Rγ: Interleukin-2 receptor γ subunit
IOD: Integrated optical density
IP: Immunoprecipitation
LSP: Long signal peptide
MLC2: Myosin light chain 2
NCG: NOD/ShiLtJGpt-Prkdc^em26Cd52^Il2rg^em26Cd22^/Gpt
NSCLC: Non-small cell lung cancer
ORF: Open reading frame
PBMCs: Peripheral blood mononuclear cells
PBS: Phosphate buffer saline
RT‒qPCR: Reverse transcription-quantitative PCR
SDs: Standard deviations
ShRNA: Short hairpin RNA
siRNA: Small interfering RNA
SSP: Short signal peptide
STR: Short tandem repeat
TILs: Tumor-infiltrating lymphocytes
TNM: Tumor-node-metastasis
WHO: World Health Organization

## References

[1] Horvath L, Thienpont B, Zhao L, Wolf D, Pircher A (2020). Overcoming immunotherapy resistance in non-small cell lung cancer (NSCLC) - novel approaches and future outlook. Mol Cancer.

[2] Martinez-Usatorre A, Kadioglu E, Boivin G, Cianciaruso C, Guichard A, Torchia B et al. Overcoming microenvironmental resistance to PD-1 blockade in genetically engineered lung cancer models. Sci Transl Med. 2021;13(606). 10.1126/scitranslmed.abd1616 PMC7612153 34380768

[3] Dong C (2021). Cytokine regulation and function in T cells. Annu Rev Immunol.

[4] Zhang Y, Guan XY, Jiang P (2020). Cytokine and chemokine signals of T-Cell exclusion in tumors. Front Immunol.

[5] Zhou X, Yu J, Cheng X, Zhao B, Manyam GC, Zhang L (2019). The deubiquitinase Otub1 controls the activation of CD8(+) T cells and NK cells by regulating IL-15-mediated priming. Nat Immunol.

[6] Hangasky JA, Chen W, Dubois SP, Daenthanasanmak A, Müller JR, Reid R et al. A very long-acting IL-15: implications for the immunotherapy of cancer. J Immunother Cancer. 2022;10(1). 10.1136/jitc-2021-004104 PMC8804710 35101947

[7] Leem G, Jeon M, Kim KW, Jeong S, Choi SJ, Lee YJ (2022). Tumour-infiltrating bystander CD8(+) T cells activated by IL-15 contribute to tumour control in non-small cell lung cancer. Thorax.

[8] Peng Y, Fu S, Zhao Q (2022). 2022 update on the scientific premise and clinical trials for IL-15 agonists as cancer immunotherapy. J Leukoc Biol.

[9] Xu H, Buhtoiarov IN, Guo H, Cheung NV (2021). A novel multimeric IL15/IL15Rα-Fc complex to enhance cancer immunotherapy. Oncoimmunology.

[10] Xu Y, Carrascosa LC, Yeung YA, Chu ML, Yang W, Djuretic I (2021). An Engineered IL15 Cytokine Mutein Fused to an Anti-PD1 improves Intratumoral T-cell function and Antitumor Immunity. Cancer Immunol Res.

[11] Pagliari D, Cianci R, Frosali S, Landolfi R, Cammarota G, Newton EE (2013). The role of IL-15 in gastrointestinal diseases: a bridge between innate and adaptive immune response. Cytokine Growth Factor Rev.

[12] Isvoranu G, Surcel M, Munteanu AN, Bratu OG, Ionita-Radu F, Neagu MT (2021). Therapeutic potential of interleukin-15 in cancer (review). Experimental Therapeutic Med.

[13] Wang X, Zhao XY (2021). Transcription factors Associated with IL-15 Cytokine Signaling during NK Cell Development. Front Immunol.

[14] Giri JG, Kumaki S, Ahdieh M, Friend DJ, Loomis A, Shanebeck K (1995). Identification and cloning of a novel IL-15 binding protein that is structurally related to the alpha chain of the IL-2 receptor. EMBO J.

[15] Mishra A, Sullivan L, Caligiuri MA (2014). Molecular pathways: interleukin-15 signaling in health and in cancer. Clin cancer Research: Official J Am Association Cancer Res.

[16] Fiore PF, Di Matteo S, Tumino N, Mariotti FR, Pietra G, Ottonello S et al. Interleukin-15 and cancer: some solved and many unsolved questions. J Immunother Cancer. 2020;8(2). 10.1136/jitc-2020-001428 PMC7674108 33203664

[17] Huang B, Liu R, Wang P, Yuan Z, Yang J, Xiong H et al. CD8(+)CD57(+) T cells exhibit distinct features in human non-small cell lung cancer. J Immunother Cancer. 2020;8(1). 10.1136/jitc-2020-000639 PMC7328901 32606053

[18] Kuniyasu H, Ohmori H, Sasaki T, Sasahira T, Yoshida K, Kitadai Y (2003). Production of interleukin 15 by human colon cancer cells is associated with induction of mucosal hyperplasia, angiogenesis, and metastasis. Clin cancer Research: Official J Am Association Cancer Res.

[19] Rohena-Rivera K, Sánchez-Vázquez MM, Aponte-Colón DA, Forestier-Román IS, Quintero-Aguiló ME, Martínez-Ferrer M (2017). IL-15 regulates migration, invasion, angiogenesis and genes associated with lipid metabolism and inflammation in prostate cancer. PLoS ONE.

[20] Khawam K, Giron-Michel J, Gu Y, Perier A, Giuliani M, Caignard A (2009). Human renal cancer cells express a novel membrane-bound interleukin-15 that induces, in response to the soluble interleukin-15 receptor alpha chain, epithelial-to-mesenchymal transition. Cancer Res.

[21] Bakir B, Chiarella AM, Pitarresi JR, Rustgi AK (2020). EMT, MET, plasticity, and Tumor Metastasis. Trends Cell Biol.

[22] Kisoda S, Mouri Y, Kitamura N, Yamamoto T, Miyoshi K, Kudo Y (2022). The role of partial-EMT in the progression of head and neck squamous cell carcinoma. J Oral Biosci.

[23] Aggarwal V, Montoya CA, Donnenberg VS, Sant S (2021). Interplay between tumor microenvironment and partial EMT as the driver of tumor progression. iScience.

[24] Brabletz S, Schuhwerk H, Brabletz T, Stemmler MP (2021). Dynamic EMT: a multi-tool for tumor progression. EMBO J.

[25] Saxena K, Jolly MK, Balamurugan K (2020). Hypoxia, partial EMT and collective migration: emerging culprits in metastasis. Translational Oncol.

[26] Jacquemet G, Hamidi H, Ivaska J (2015). Filopodia in cell adhesion, 3D migration and cancer cell invasion. Curr Opin Cell Biol.

[27] Tong J, Li L, Ballermann B, Wang Z (2016). Phosphorylation and activation of RhoA by ERK in response to epidermal growth factor stimulation. PLoS ONE.

[28] Haslene-Hox H, Oveland E, Berg KC, Kolmannskog O, Woie K, Salvesen HB (2011). A new method for isolation of interstitial fluid from human solid tumors applied to proteomic analysis of ovarian carcinoma tissue. PLoS ONE.

[29] Patidar M, Yadav N, Dalai SK (2016). Interleukin 15: a key cytokine for immunotherapy. Cytokine Growth Factor Rev.

[30] Kwon KW, Kim SJ, Kim H, Kim WS, Kang SM, Choi E (2019). IL-15 generates IFN-γ-producing cells reciprocally expressing lymphoid-myeloid markers during dendritic cell differentiation. Int J Biol Sci.

[31] Trinder P, Seitzer U, Gerdes J, Seliger B, Maeurer M (1999). Constitutive and IFN-gamma regulated expression of IL-7 and IL-15 in human renal cell cancer. Int J Oncol.

[32] Kansler ER, Dadi S, Krishna C, Nixon BG, Stamatiades EG, Liu M (2022). Cytotoxic innate lymphoid cells sense cancer cell-expressed interleukin-15 to suppress human and murine malignancies. Nat Immunol.

[33] Zhou Y, Husman T, Cen X, Tsao T, Brown J, Bajpai A et al. Interleukin 15 in cell-based Cancer Immunotherapy. Int J Mol Sci. 2022;23(13). 10.3390/ijms23137311 PMC9266896 35806311

[34] Kobayashi H, Gotoh J, Fujie M, Shinohara H, Moniwa N, Terao T (1994). Inhibition of metastasis of Lewis lung carcinoma by a synthetic peptide within growth factor-like domain of urokinase in the experimental and spontaneous metastasis model. Int J Cancer.

[35] Zhao M, Suetsugu A, Ma H, Zhang L, Liu F, Zhang Y (2012). Efficacy against lung metastasis with a tumor-targeting mutant of Salmonella typhimurium in immunocompetent mice. Cell Cycle (Georgetown Tex).

[36] Alhakamy NA, Ishiguro S, Uppalapati D, Berkland CJ, Tamura M (2016). AT2R gene delivered by condensed polylysine complexes attenuates Lewis Lung Carcinoma after Intravenous injection or Intratracheal Spray. Mol Cancer Ther.

[37] Andrews MG, Subramanian L, Kriegstein AR. mTOR signaling regulates the morphology and migration of outer radial glia in developing human cortex. eLife. 2020;9. 10.7554/eLife.58737 PMC7467727 32876565

[38] Krugmann S, Jordens I, Gevaert K, Driessens M, Vandekerckhove J, Hall A (2001). Cdc42 induces filopodia by promoting the formation of an IRSp53:Mena complex. Curr Biology: CB.

[39] Kidd ME, Shumaker DK, Ridge KM (2014). The role of vimentin intermediate filaments in the progression of lung cancer. Am J Respir Cell Mol Biol.

[40] Dongre A, Weinberg RA (2019). New insights into the mechanisms of epithelial-mesenchymal transition and implications for cancer. Nat Rev Mol Cell Biol.

[41] Sengupta S, Lohse CM, Leibovich BC, Frank I, Thompson RH, Webster WS (2005). Histologic coagulative tumor necrosis as a prognostic indicator of renal cell carcinoma aggressiveness. Cancer.

[42] Kurys G, Tagaya Y, Bamford R, Hanover JA, Waldmann TA (2000). The long signal peptide isoform and its alternative processing direct the intracellular trafficking of interleukin-15. J Biol Chem.

[43] Bergamaschi C, Jalah R, Kulkarni V, Rosati M, Zhang GM, Alicea C et al. Secretion and biological activity of short signal peptide IL-15 is chaperoned by IL-15 receptor alpha in vivo. Journal of immunology (Baltimore, Md: 1950). 2009;183(5):3064-72. 10.4049/jimmunol.0900693 PMC7250482 19696432

[44] Amano M, Chihara K, Kimura K, Fukata Y, Nakamura N, Matsuura Y (1997). Formation of actin stress fibers and focal adhesions enhanced by rho-kinase.

[45] Katoh K, Kano Y, Amano M, Onishi H, Kaibuchi K, Fujiwara K (2001). Rho-kinase–mediated contraction of isolated stress fibers. J Cell Biol.

[46] Tkach V, Bock E, Berezin V (2005). The role of RhoA in the regulation of cell morphology and motility. Cell Motil Cytoskeleton.

[47] Duan X, Liu J, Zhu CC, Wang QC, Cui XS, Kim NH (2016). RhoA-mediated MLC2 regulates actin dynamics for cytokinesis in meiosis. Cell Cycle (Georgetown Tex).

[48] Sharanek A, Burban A, Burbank M, Le Guevel R, Li R, Guillouzo A (2016). Rho-kinase/myosin light chain kinase pathway plays a key role in the impairment of bile canaliculi dynamics induced by cholestatic drugs. Sci Rep.

[49] Chrzanowska-Wodnicka M, Burridge K (1996). Rho-stimulated contractility drives the formation of stress fibers and focal adhesions. J Cell Biol.

[50] Zhang H, Schaefer A, Wang Y, Hodge RG, Blake DR, Diehl JN (2020). Gain-of-function RHOA mutations promote focal adhesion kinase activation and dependency in diffuse gastric Cancer. Cancer Discov.

[51] Sato N, Patel HJ, Waldmann TA, Tagaya Y (2007). The IL-15/IL-15Ralpha on cell surfaces enables sustained IL-15 activity and contributes to the long survival of CD8 memory T cells. Proc Natl Acad Sci USA.

[52] Mortier E, Woo T, Advincula R, Gozalo S, Ma A (2008). IL-15Ralpha chaperones IL-15 to stable dendritic cell membrane complexes that activate NK cells via trans presentation. J Exp Med.

[53] Hasan AN, Selvakumar A, Shabrova E, Liu XR, Afridi F, Heller G (2016). Soluble and membrane-bound interleukin (IL)-15 Rα/IL-15 complexes mediate proliferation of high-avidity central memory CD8(+) T cells for adoptive immunotherapy of cancer and infections. Clin Exp Immunol.

[54] Bosch NC, Martin LM, Voskens CJ, Berking C, Seliger B, Schuler G et al. A Chimeric IL-15/IL-15Rα Molecule Expressed on NFκB-Activated Dendritic Cells Supports Their Capability to Activate Natural Killer Cells. International journal of molecular sciences. 2021;22(19). 10.3390/ijms221910227 PMC8508776 34638566

[55] Wang W, Zhu L, Zhou J, Liu X, Xiao M, Chen N (2023). Targeting the KRT16-vimentin axis for metastasis in lung cancer. Pharmacol Res.

[56] Gallop JL (2020). Filopodia and their links with membrane traffic and cell adhesion. Semin Cell Dev Biol.

[57] Iseppon F, Napolitano LM, Torre V, Cojoc D (2015). Cdc42 and RhoA reveal different spatio-temporal dynamics upon local stimulation with Semaphorin-3A. Front Cell Neurosci.

[58] He K, Sakai T, Tsukasaki Y, Watanabe TM, Ikebe M (2017). Myosin X is recruited to nascent focal adhesions at the leading edge and induces multi-cycle filopodial elongation. Sci Rep.

[59] Anderson DM, Kumaki S, Ahdieh M, Bertles J, Tometsko M, Loomis A (1995). Functional characterization of the human interleukin-15 receptor alpha chain and close linkage of IL15RA and IL2RA genes. J Biol Chem.

[60] Pereno R, Giron-Michel J, Gaggero A, Cazes E, Meazza R, Monetti M (2000). IL-15/IL-15Ralpha intracellular trafficking in human melanoma cells and signal transduction through the IL-15Ralpha. Oncogene.

[61] Perdreau H, Mortier E, Bouchaud G, Solé V, Boublik Y, Plet A (2010). Different dynamics of IL-15R activation following IL-15 cis- or trans-presentation. Eur Cytokine Netw.

[62] Zanoni I, Spreafico R, Bodio C, Di Gioia M, Cigni C, Broggi A (2013). IL-15 cis presentation is required for optimal NK cell activation in lipopolysaccharide-mediated inflammatory conditions. Cell Rep.

[63] Varadarajan N, Mohan C (2019). Elucidating the molecular circuitry of autoimmunity. Nat Immunol.

[64] Stonier SW, Schluns KS (2010). Trans-presentation: a novel mechanism regulating IL-15 delivery and responses. Immunol Lett.

[65] Tagaya Y, Kurys G, Thies TA, Losi JM, Azimi N, Hanover JA (1997). Generation of secretable and nonsecretable interleukin 15 isoforms through alternate usage of signal peptides. Proc Natl Acad Sci USA.

[66] Meazza R, Gaggero A, Neglia F, Basso S, Sforzini S, Pereno R (1997). Expression of two interleukin-15 mRNA isoforms in human tumors does not correlate with secretion: role of different signal peptides. Eur J Immunol.

[67] Bamford RN, Battiata AP, Burton JD, Sharma H, Waldmann TA, Interleukin (1996). IL) 15/IL-T production by the adult T-cell leukemia cell line HuT-102 is associated with a human T-cell lymphotrophic virus type I region /IL-15 fusion message that lacks many upstream AUGs that normally attenuates IL-15 mRNA translation. Proc Natl Acad Sci USA.

[68] Waldmann TA, Tagaya Y (1999). The multifaceted regulation of interleukin-15 expression and the role of this cytokine in NK cell differentiation and host response to intracellular pathogens. Annu Rev Immunol.

[69] Bamford RN, DeFilippis AP, Azimi N, Kurys G, Waldmann TA. The 5’ untranslated region, signal peptide, and the coding sequence of the carboxyl terminus of IL-15 participate in its multifaceted translational control. Journal of immunology (Baltimore, Md: 1950). 1998;160(9):4418-26. 9574546

[70] Jabri B, Abadie V (2015). IL-15 functions as a danger signal to regulate tissue-resident T cells and tissue destruction. Nat Rev Immunol.

[71] Araki A, Hazama S, Yoshimura K, Yoshino S, Iizuka N, Oka M (2004). Tumor secreting high levels of IL-15 induces specific immunity to low immunogenic colon adenocarcinoma via CD8 + T cells. Int J Mol Med.

[72] Mortier E, Quéméner A, Vusio P, Lorenzen I, Boublik Y, Grötzinger J (2006). Soluble interleukin-15 receptor alpha (IL-15R alpha)-sushi as a selective and potent agonist of IL-15 action through IL-15R beta/gamma. Hyperagonist IL-15 x IL-15R alpha fusion proteins. J Biol Chem.

[73] Giron-Michel J, Giuliani M, Fogli M, Brouty-Boyé D, Ferrini S, Baychelier F (2005). Membrane-bound and soluble IL-15/IL-15Ralpha complexes display differential signaling and functions on human hematopoietic progenitors. Blood.

[74] Ellis-Connell AL, Balgeman AJ, Zarbock KR, Barry G, Weiler A, Egan JO et al. ALT-803 transiently reduces simian immunodeficiency virus replication in the absence of antiretroviral treatment. J Virol. 2018;92(3). 10.1128/JVI.01748-17 PMC5774892 29118125

[75] Wrangle JM, Velcheti V, Patel MR, Garrett-Mayer E, Hill EG, Ravenel JG (2018). ALT-803, an IL-15 superagonist, in combination with nivolumab in patients with metastatic non-small cell lung cancer: a non-randomised, open-label, phase 1b trial. Lancet Oncol.

