# Supplementary material for: Lung cancer cell-intrinsic IL-15 promotes cell migration and sensitizes murine lung tumors to anti-PD-L1 therapy
Source: Biomark Res. 2024 Apr 19;12:40. doi: 10.1186/s40364-024-00586-w (PMC11027539; doi:10.1186/s40364-024-00586-w)
Supplement: Supplementary file 1 — Supplementary Material 1 [file 40364_2024_586_MOESM1_ESM.docx]

|  | **Table S1. List of Antibodies and reagents** | | | | | | |
| --- | --- | --- | --- | --- | --- | --- | --- |
|  | **Antibodies** | | **Clone** | **Applications** | **Supplier** | **Catalog Number** | **RRIDs** |
|  | anti IL15 | | 34593 | WB | R＆D | MAB247 | AB_2124578 |
|  | anti-IL2Rβ | | polyclone | WB | Abcam | ab61195 | AB_943560 |
|  | anti IL15 | | monoclonal | WB/IP/IHC | Santa Cruz Biotechnology | sc-8437 | AB_2124575 |
|  | anti IL15Ra | | monoclonal | WB/IP | Santa Cruz Biotechnology | sc-374023 | AB_10917901 |
|  | anti IL15Ra | | polyclone | WB | R＆D | AF247 | AB_355274 |
|  | anti IL2Rγ | | polyclone | WB | R＆D | AF284 | AB_355292 |
|  | anti-IL2Rβ | | monoclonal | WB | Santa Cruz Biotechnology | sc-393093 | - |
|  | anti IL2Rγ | | monoclonal | WB | Santa Cruz Biotechnology | sc-271060 | AB_10614502 |
|  | anti Mouse IL-15 | | polyclone | WB | R＆D | AF447 | AB_2124437 |
|  | anti p-stat5 | | monoclonal | WB | Cell Signaling Technologies | 4322 | AB_10544692 |
|  | anti stat5 | | polyclone | WB | Cell Signaling Technologies | 9363 | AB_2196923 |
|  | anti β-actin | | 8H10D10 | WB | Cell Signaling Technologies | 3700 | AB_2242334 |
|  | anti vinculin | | monoclonal | IF | proteintech | 26520-1-AP | AB_2868558 |
|  | anti-GAPDH | | monoclonal | WB | proteintech | 60004-1-Ig | AB_2107436 |
|  | anti CD8α | | recombinant monoclonal | IHC | Cell Signaling Technologies | 98941 | AB_2756376 |
|  | anti p-MLC2 | | polyclone | WB | Cell Signaling Technologies | 3674 | AB_2147464 |
|  | MLC2 | | Polyclonal | WB | proteintech | 10906-1-AP | AB_2147453 |
|  | anti RHOA | | monoclonal | WB | Cell Signaling Technologies | 2117 | AB_10693922 |
|  | anti-ZEB1 | | D80D3 | WB | Cell Signaling Technologies | 3396 | AB_1904164 |
|  | anti-E-cadherin | | 2.4E+11 | WB | Cell Signaling Technologies | 3195 | AB_2291471 |
|  | anti-Vimentin | | D21H3 | WB | Cell Signaling Technologies | 5741 | AB_10695459 |
|  | anti-snail | | C15D3 | WB | Cell Signaling Technologies | 3879 | AB_2255011 |
|  | anti-slug | | C19G7 | WB | Cell Signaling Technologies | 9585 | AB_2239535 |
|  | anti p-Akt | | D9E | WB | Cell Signaling Technologies | 4060 | AB_2315049 |
|  | anti AKT | | 40D4 | WB | Cell Signaling Technologies | 2920 | AB_1147620 |
|  | anti p-ERK1/2 | | D13.14.4E | WB | Cell Signaling Technologies | 4370 | AB_2315112 |
|  | anti ERK1/2 | | 137F5 | WB | Cell Signaling Technologies | 4695 | AB_390779 |
|  | anti p-FAK | | monoclonal | WB | Cell Signaling Technologies | 8556 | AB_10891442 |
|  | anti p-S6 | | monoclonal | WB | Cell Signaling Technologies | 4858 | AB_916156 |
|  | anti S6 | | polyclonal | WB | Proteintech | 14823-1-AP | AB_2181025 |
|  | anti p-P70S6K | | monoclonal | WB | Cell Signaling Technologies | 9234 | AB_2269803 |
|  | anti P70S6K | | monoclonal | WB | Cell Signaling Technologies | 2708 | AB_390722 |
|  | HRP, goat anti mouse IgG | | polyclone | WB | Abbkine | A21010 | AB_2728771 |
|  | HRP, goat anti rabbit IgG | | polyclone | WB | Abbkine | A21020 | AB_2876889 |
|  | HRP, donkey anti goat IgG | | polyclone | WB | Servicebio | GB23404 | - |
|  | FITC conjugated Goat Anti-Rabbit IgG | | polyclone | IF | Servicebio | GB22303 | - |
|  | Cy5 conjugated Goat Anti-rabbit IgG | | polyclone | IF | Servicebio | GB27303 | - |
|  | Anti-mouse PD-L1 | | 10F.9G2 | neutralization | bioxcell | BE0101 | - |
|  | IgG2b isotype control | | LTF-2 | neutralization | bioxcell | BE0090 | - |
|  | **Antibodies** | | **Clone** | **Applications** | **Supplier** | **Catalog Number** | **RRIDs** |
|  | BV510 anti-mouse CD8 | | 53-6.7 | flow | biolegend | 100751 | AB_2561389 |
|  | PE anti-mouse CD3 | | 17A2 | flow | biolegend | 100205 | AB_312662 |
|  | FITC anti-mouse GZMB | | GB11 | flow | biolegend | 515403 | AB_2114575 |
|  | BV421 anti-mouse CD45 | | 30-F11 | flow | biolegend | 103133 | AB_10899570 |
|  | PEcy7 anti-mouse Ki-67 | | 16A8 | flow | biolegend | 652426 | AB_2632694 |
|  | APC anti-mouse perforin | | S16009A | flow | biolegend | 154303 | AB_2721462 |
|  | BV711 Anti-Human CD4 antibody | | SK3 | flow | BD | 563028 | AB_2737961 |
|  | BV605 Anti-Human CD3 antibody | | SK7 | flow | BD | 563219 | AB_2714001 |
|  | PE/Cyanine7 anti-human Ki-67 | | monoclonal | flow | biolegend | 350526 | AB_2562871 |
|  | FITC anti-human Granzyme B | | monoclonal | flow | biolegend | 515403 | AB_2114575 |
|  | APC anti-human Perforin | | monoclonal | flow | biolegend | 353312 | AB_2571968 |
|  | **Reagents** | | **Supplier** | **Catalog Number** |  |  |  |
|  | Cell Counting Kit-8 | | Abbkine | BMU106-CN |  |  |  |
|  | Human IL15 | | biolegend | 570304 |  |  |  |
|  | RhoA pulldown activation Assay Kit | | cytoskeleton | BK036-S |  |  |  |
|  | Human IL15Ra＆IL15 Fusion protein | | MCE | HY-P70655 |  |  |  |
|  | LY294002 | | MCE | HY-10108 |  |  |  |
|  | Protein A/G Magnetic Beads | | MCE | HY-K0202 |  |  |  |
|  | iFluor™ 647 phalloidin | | Yeasen | 40762ES75 |  |  |  |
|  | Mouse lymphocyte separation medium | | DAKEWE | 7211011 |  |  |  |
|  | Fixable Viability Dye eFluor™ 780 | | eBioscience | 65-0865-14 |  |  |  |
|  | Corning Matrigel matrix | | Corning | 354234 |  |  |  |
|  | IL15 Mouse elisa development kit | | Invitrogen | 900-K188 |  |  |  |
|  | Human lymphocyte separation medium | | DAKEWE | 7111012 |  |  |  |
|  | Rapamycin | | MCE | AY-22989 |  |  |  |
|  | Cdc42 activation assay kit | | New East Biosciences | 80701 |  |  |  |
|  | Cycloheximide | | MCE | HY-12320 |  |  |  |
|  |  |  |  |  |  |  |  |
