# Supplementary figures and images for: Lung cancer cell-intrinsic IL-15 promotes cell migration and sensitizes murine lung tumors to anti-PD-L1 therapy
Source: Biomark Res. 2024 Apr 19;12:40. doi: 10.1186/s40364-024-00586-w (PMC11027539; doi:10.1186/s40364-024-00586-w)

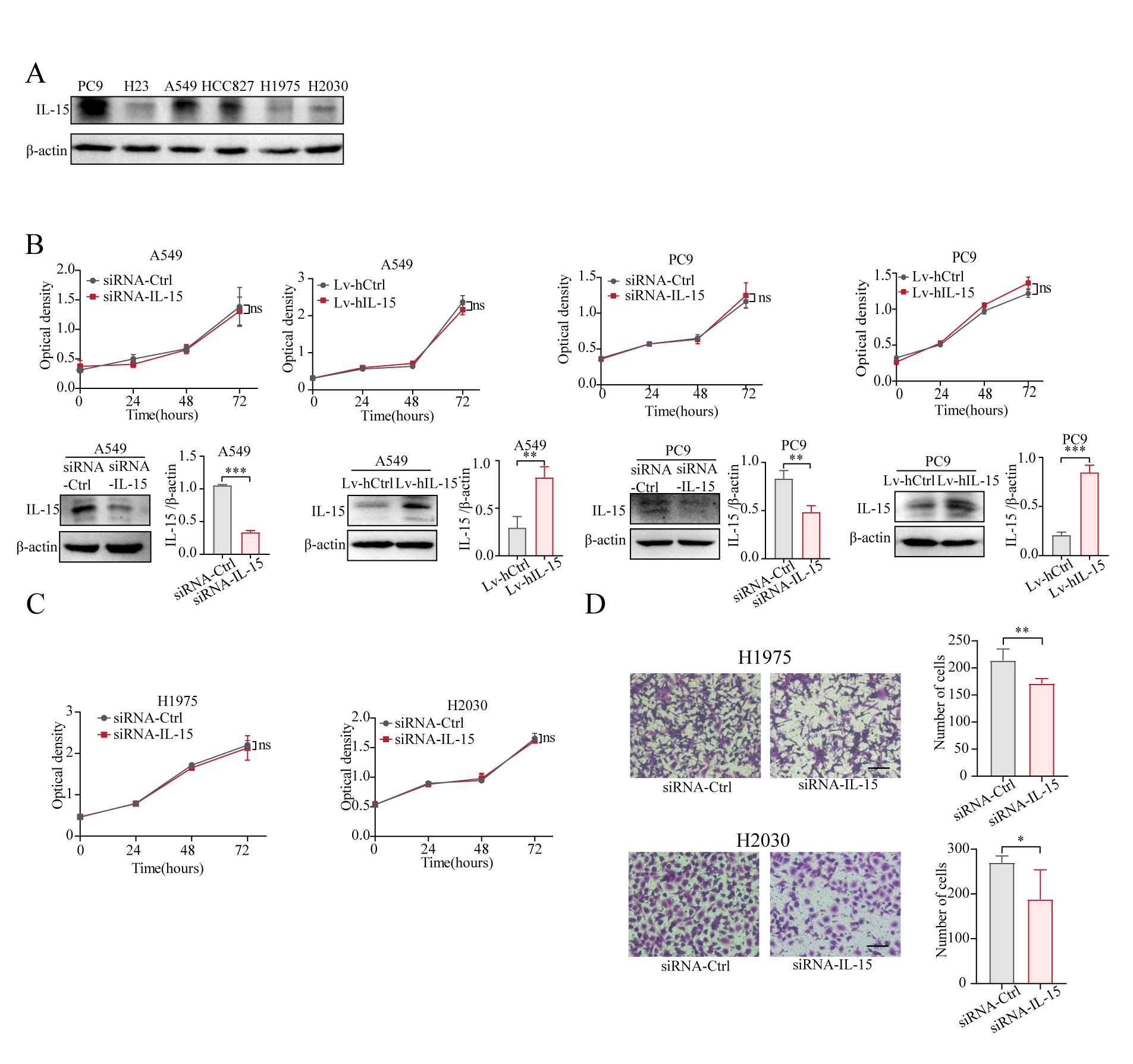

Supplement: Supplementary file 3 — Supplementary Material 3 [file 40364_2024_586_MOESM3_ESM.png]

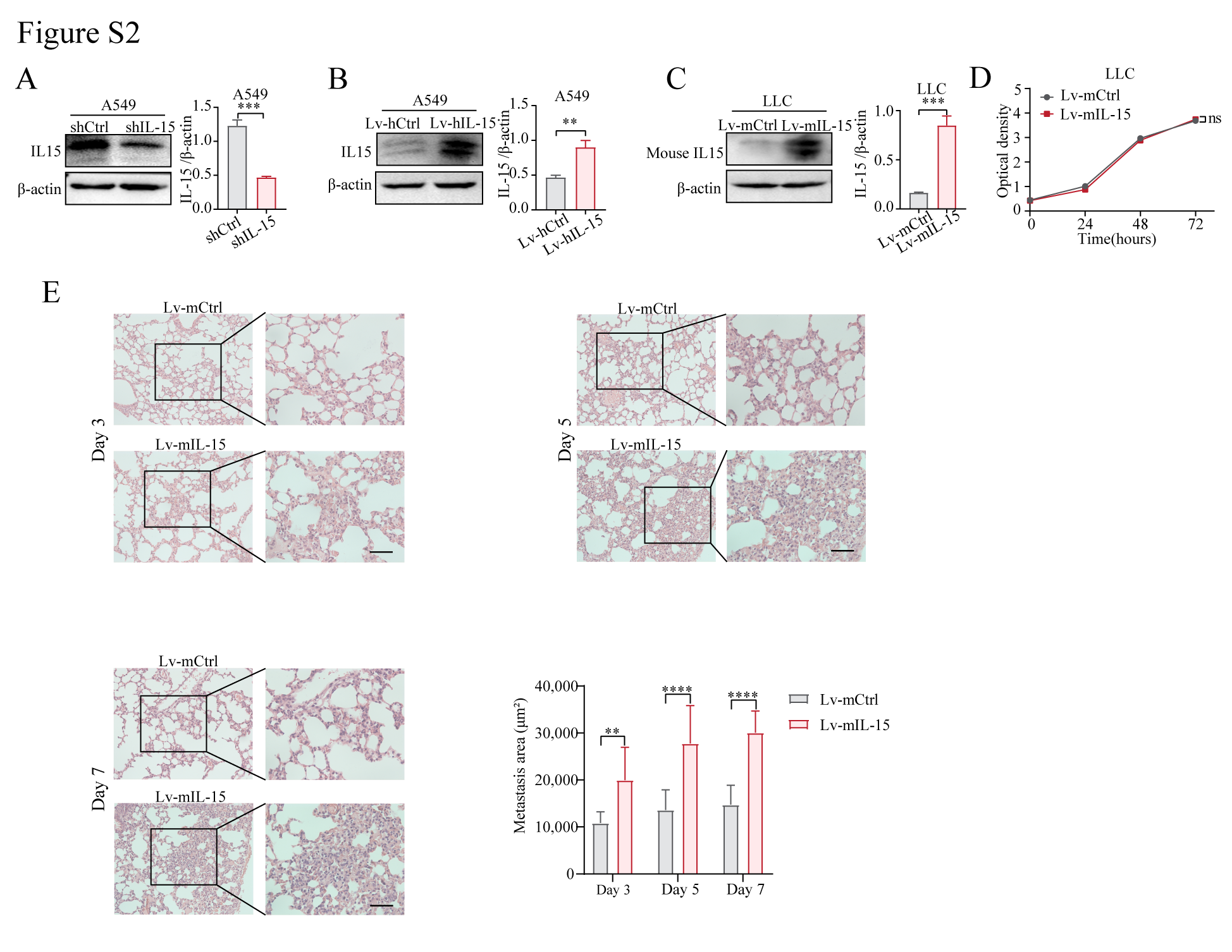

Supplement: Supplementary file 4 — Supplementary Material 4 [file 40364_2024_586_MOESM4_ESM.png]

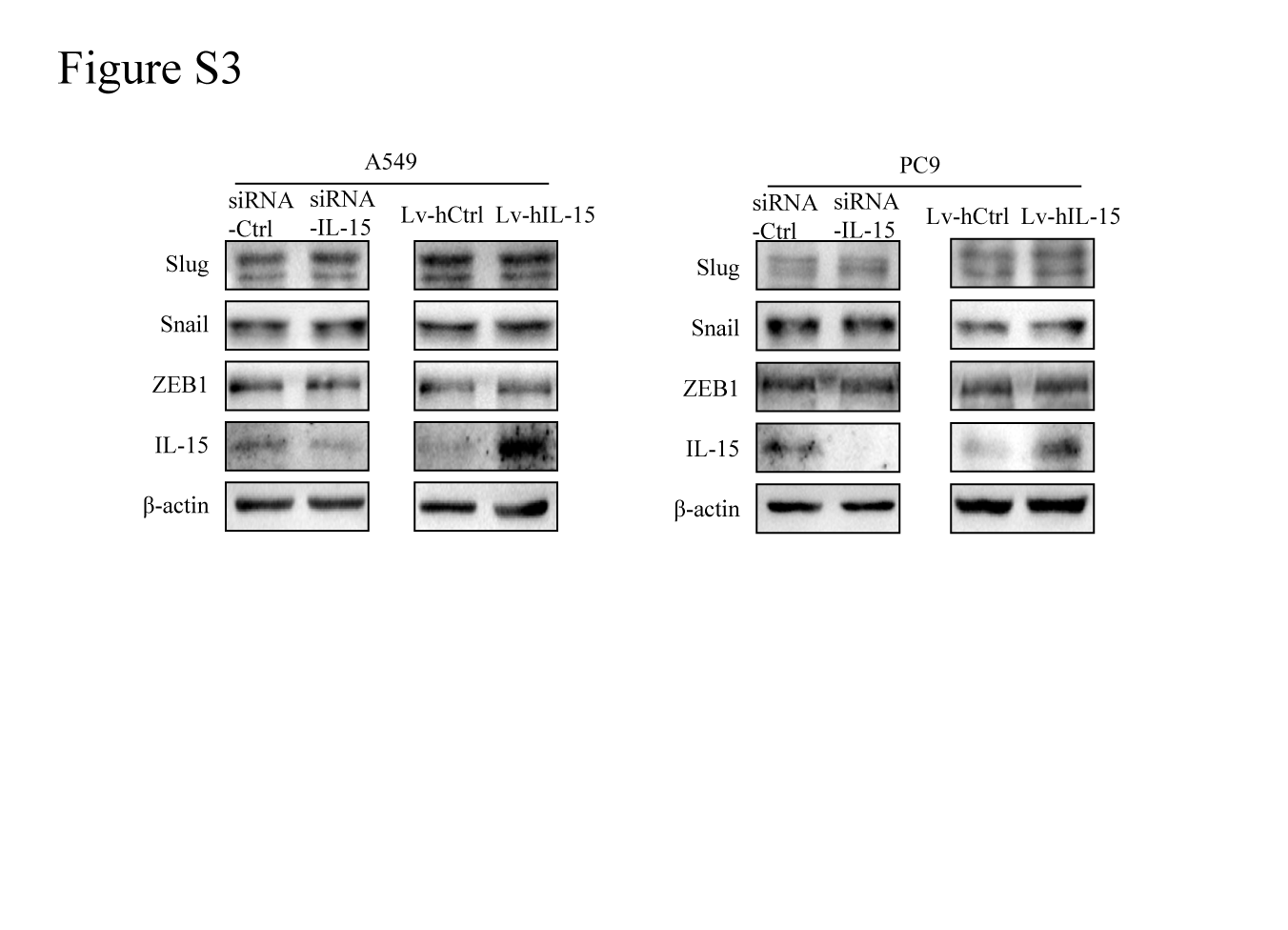

Supplement: Supplementary file 5 — Supplementary Material 5 [file 40364_2024_586_MOESM5_ESM.png]

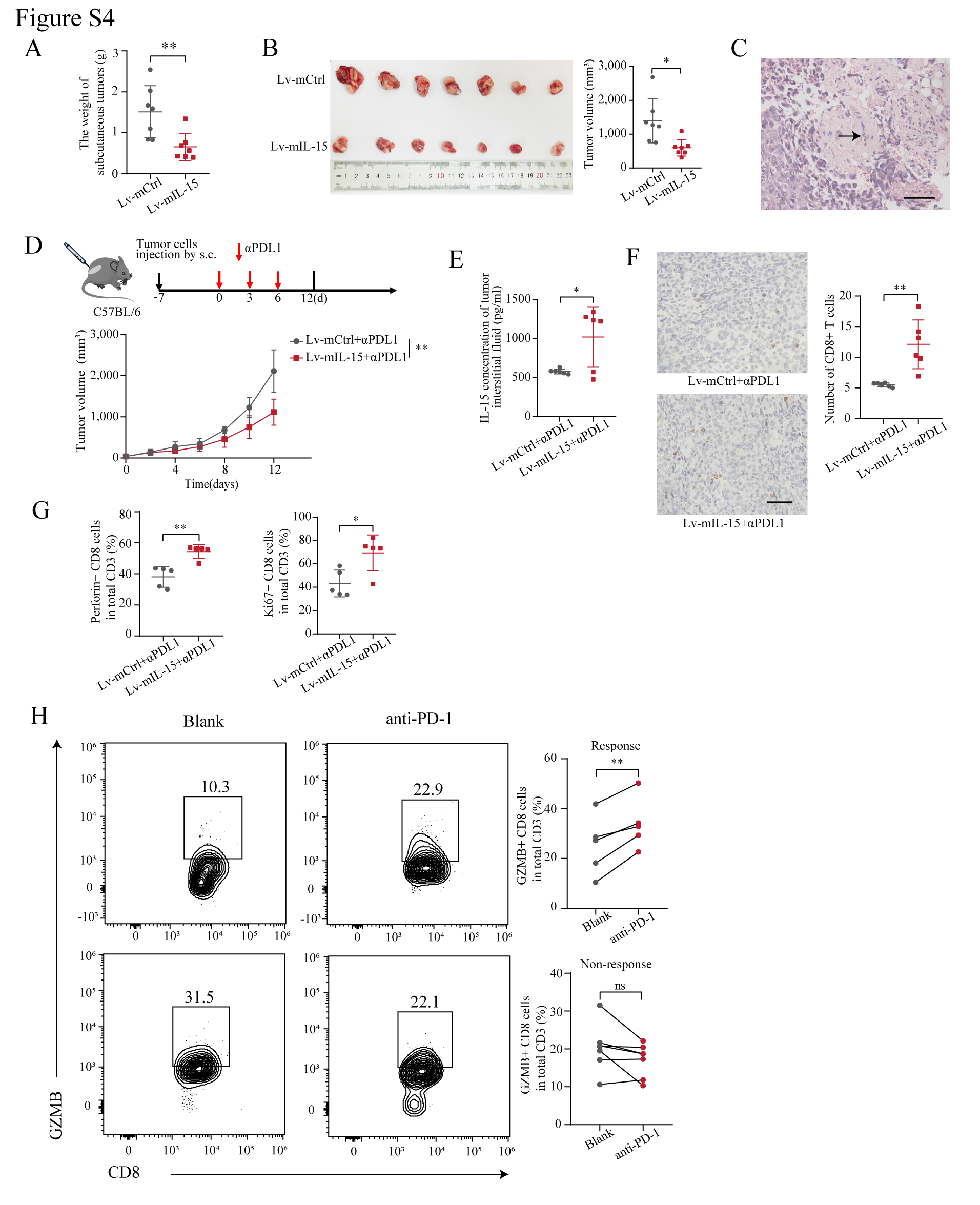

Supplement: Supplementary file 6 — Supplementary Material 6 [file 40364_2024_586_MOESM6_ESM.png]

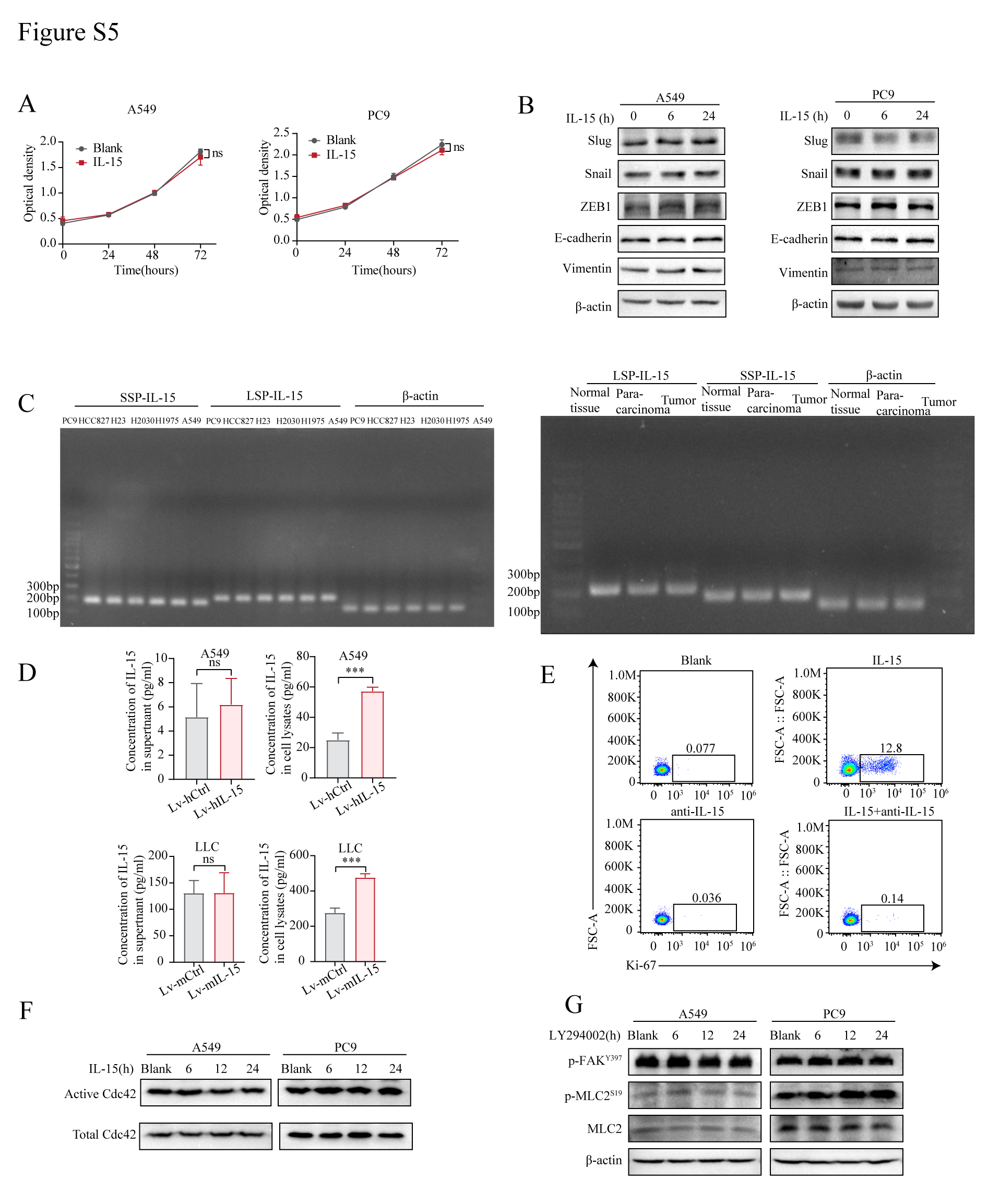

Supplement: Supplementary file 7 — Supplementary Material 7 [file 40364_2024_586_MOESM7_ESM.png]

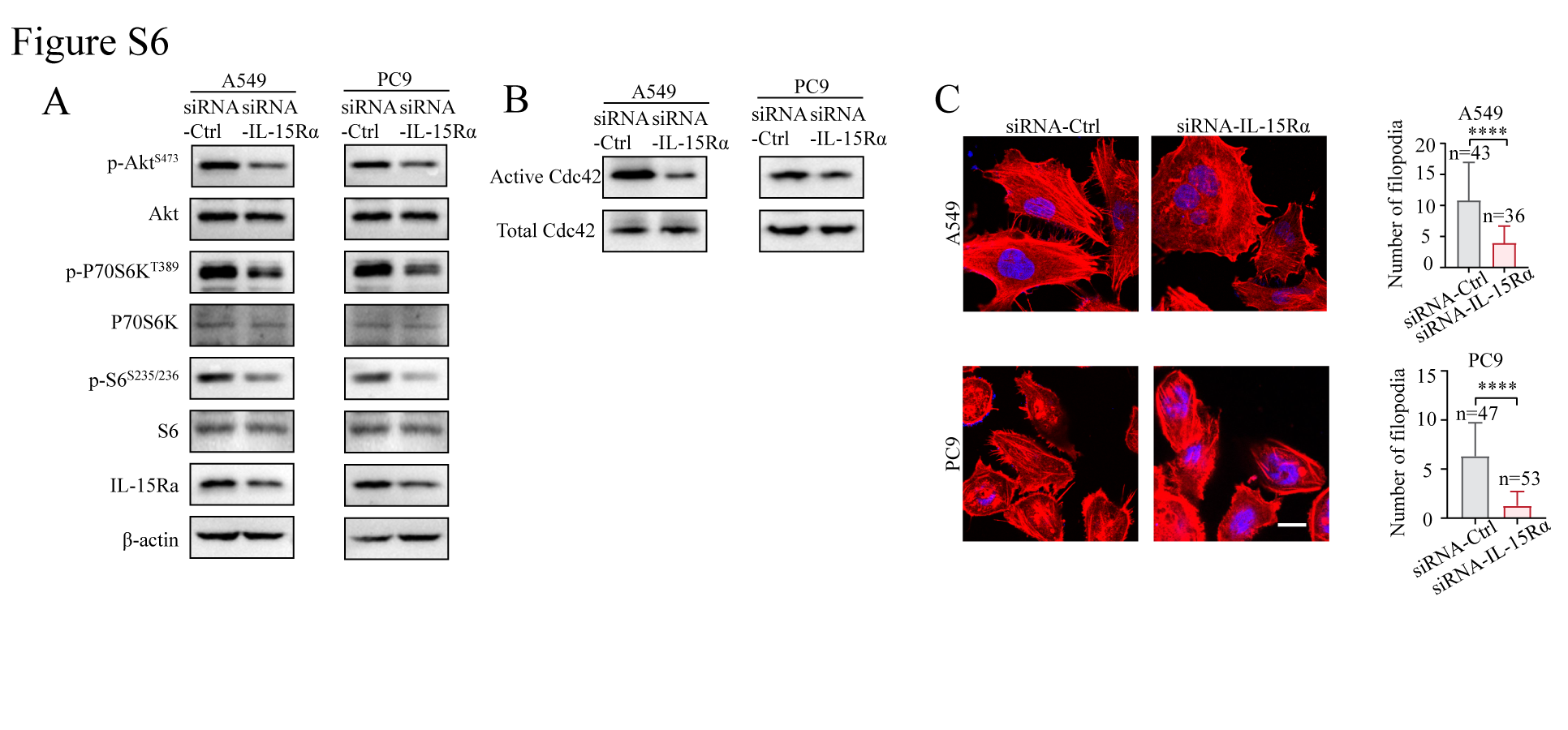

Supplement: Supplementary file 8 — Supplementary Material 8 [file 40364_2024_586_MOESM8_ESM.png]
