# Supplementary material for: Lung cancer cell-intrinsic IL-15 promotes cell migration and sensitizes murine lung tumors to anti-PD-L1 therapy
Source: Biomark Res. 2024 Apr 19;12:40. doi: 10.1186/s40364-024-00586-w (PMC11027539; doi:10.1186/s40364-024-00586-w)
Supplement: Supplementary file 9 — Supplementary Material 9 [file 40364_2024_586_MOESM9_ESM.docx]

**Figure S1 Cancer cell-intrinsic IL-15 does not affect cell proliferation but promotes cell migration.**

**A**, The expression of IL-15 in lung adenocarcinoma cell lines was analyzed by immunoblotting. **B**, A CCK8 assay was performed on A549 and PC9 cells transfected with siRNA-IL15 and siRNA-Ctrl or transduced with Lv-hIL-15 and Lv-hCtrl. The bar graph represents the expression of IL-15 relative to that of β-actin, and Image J software was used. The data are presented as the means ± SDs and were analyzed by two-tailed Student’s *t* test. **C**, A CCK8 assay was performed on H1975 and H2030 cells transfected with siRNA-IL15 and siRNA-Ctrl. **D**, A Transwell migration assay was performed on H1975 and H2030 cells transfected with siRNA-IL15 and siRNA-Ctrl. Scale bar, 100 μm. *, *p* < 0.05; **, *p* < 0.01.

**Figure S2 Cancer cell-intrinsic IL-15 facilitates the formation of metastases *in vivo*.**

**A-C**, Transfection efficiency was assessed by immunoblot analysis, and Image J software was used to analyze the expression of IL-15 relative to that of β-actin. The data are presented as the means ± SDs and were analyzed by two-tailed Student’s *t* test. **D**, A CCK-8 assay was performed on LLC cells transfected with Lv-mIL-15 or Lv-mCtrl. **E**, IL-15-overexpressing LLC cells and Lv-Ctrl LLC cells were injected into C57BL/6 mice via the tail vein (1×10^6^ cells/mouse, n=2). The mice were sacrificed on days 3, 5, and 7 after the injection of cells. Lung metastasis was evaluated by IHC staining. The data are presented as the means ± SDs. Scale bar, 50 μm. **, *p* < 0.01; ****, *p* < 0.0001.

**Figure S3 Effect of cancer cell-intrinsic IL-15 on EMT-associated transcription factors.**

The expression of the indicated proteins was analyzed by immunoblotting. β-Actin was used as the protein loading control.

**Figure S4 Cancer cell-intrinsic IL-15 enhances the efficacy of immunotherapy**

**A,** The experiments were conducted as described in Figure 5C. LLC cells transduced with Lv-mIL-15 or Lv-mCtrl were inoculated subcutaneously into C57BL/6 mice (1 × 10^6^ cells/mouse, n = 7 mice/group). Tumor weight was measured 19 days after inoculation. The data are presented as the means ± SDs and were analyzed by two-tailed Student’s *t* test. **B**, The tumor gross images and volume are displayed (n=7). The data are presented as the means ± SDs and were analyzed by two-tailed Student’s *t* test. **C**, The subcutaneous tumor tissues were fixed, sectioned, and observed under a light microscope after hematoxylin and eosin staining. The black arrow indicates nucleus fragments. Scale bar, 50 μm. **D**, LLC cells transduced with Lv-mIL-15 or Lv-mCtrl were inoculated subcutaneously into C57BL/6 mice (1 × 10^6^ cells/mouse, n = 7 mice/group). Seven days after the subcutaneous injection of LLC cells into the mice, both groups were treated with an anti-PD-L1 antibody ((i.p., 10 mg/kg, day 0). Tumor volumes were calculated, and the data are presented as the means ± SDs. Two-way ANOVA followed by Sidak’s multiple comparison test was used. **, *p* < 0.01. **E**, The concentration of IL-15 in the tumor interstitial fluid was measured via ELISA. Each point in the graph represents an individual mouse. The data are presented as the means ± SDs. *, *p* < 0.05. **F**, Representative images of tumor-infiltrating CD8+ T cells in tumors. Scale bar, 50 μm. **, *p* < 0.01. **G**, TILs were enriched from the tumors and stained with antibodies against CD45, CD3, CD8, Ki67, and perforin. The data are presented as the means ± SDs (n = 5). *, *p* < 0.05; **, *p* < 0.01. **H**, Freshly collected human lung tumor explants were treated with 10 μg/ml anti-PD-1 for 24 h. TILs were isolated from the explants and GZMB expression on CD8+ T cells was analyzed using flow cytometry. **, *p* < 0.01.

**Figure S5 Effect of exogenous IL-15 on tumor cell proliferation, EMT and Cdc42 activity.**

**A**, A CCK-8 assay was performed on A549 and PC9 cells in the presence or absence of IL-15 (100 ng/mL). **B**, A549 and PC9 cells were cultured with IL-15 for 6 or 24 h. The cell lysates were subjected to immunoblotting to measure the expression of the indicated proteins. **C**, The expression of the two isoforms of IL-15, LSP-IL15 and SSP-IL15, in lung adenocarcinoma cell lines and tumors was measured by qPCR. **D,** The amount of IL-15 in the supernatants or cell lysates was assessed via ELISA. **E**, PBMCs were treated with IL-15 (10 ng/ml) in the presence or absence of an IL-15 neutralizing antibody (10 μg/ml) for 24 h. The level of Ki-67 expression in CD8^+^ T cells was measured by staining the cells with an antibody against Ki-67 and analyzing by flow cytometry. **F,** Cdc42 activity was evaluated in A549 and PC9 cells treated with IL-15 (100 ng/ml) for 6, 12, or 24 h. **G,** The phosphorylation levels of FAK and MLC2a in A549 and PC9 cells treated with the PI3K inhibitor LY294002 (5 μM) for 6, 12, or 24 h were detected by immunoblotting.

**Figure S6 The effects of IL-15Rα on morphology and the AKT-mTORC1-Cdc42 signaling pathway.**

**A,** The phosphorylation of AKT, P70S6K, and S6 was assessed by immunoblotting after knocking down IL-15Rα in A549 and PC9 cells. **B**, Cdc42 activity was measured in A549 and PC9 cells after IL-15Rα was knocked down. **C**, The indicated cells were stained with iFluor647-labeled phalloidin for visualization of filopodia. The number of filopodia was quantified using ImageJ analysis. The data are presented as the means ± SDs and were analyzed by two-tailed Student’s t test. Scale bar, 10 μm.
